# Comparing Isoelectronic, Quadruple-Bonded Metalloporphyrin and Metallocorrole Dimers: Scalar-Relativistic DFT Calculations Predict a >1 eV Range for Ionization Potential and Electron Affinity

**DOI:** 10.1021/acsphyschemau.1c00030

**Published:** 2021-10-21

**Authors:** Jeanet Conradie, Hugo Vazquez-Lima, Abraham B. Alemayehu, Abhik Ghosh

**Affiliations:** † Department of Chemistry, UiT − The Arctic University of Norway, N-9037 Tromsø, Norway; ‡ Department of Chemistry, University of the Free State, P.O. Box 339, Bloemfontein 9300, Republic of South Africa

**Keywords:** metal−metal bond, quadruple bond, δ-bond, porphyrin, corrole, metalloporphyrin, metallocorrole

## Abstract

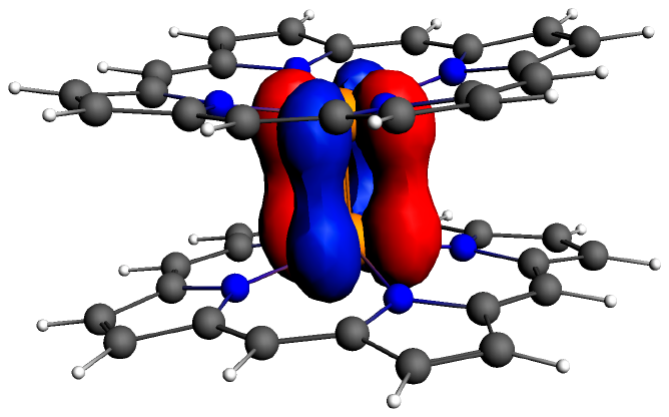

A scalar-relativistic DFT study of isoelectronic, quadruple-bonded Group 6 metalloporphyrins (M = Mo, W) and Group 7 metallocorroles (M = Tc, Re) has uncovered dramatic differences in ionization potential (IP) and electron affinity (EA) among the compounds. Thus, both the IPs and EAs of the corrole derivatives are 1 eV or more higher than those of the porphyrin derivatives. These differences largely reflect the much lower orbital energies of the δ- and δ*-orbitals of the corrole dimers relative to those of the porphyrin dimers, which in turn reflect the higher (+III as opposed to +II) oxidation states of the metals in the former compounds. Significant differences have also been determined between Mo and W porphyrin dimers and between Tc and Re corrole dimers. These differences are thought to largely reflect greater relativistic destabilization of the 5d orbitals of W and Re relative to the 4d orbitals of Mo and Tc. The calculated differences in IP and EA should translate to major differences in electrochemical redox potentials—a prediction that in our opinion is well worth confirming.

## Introduction

**Chart 1:**
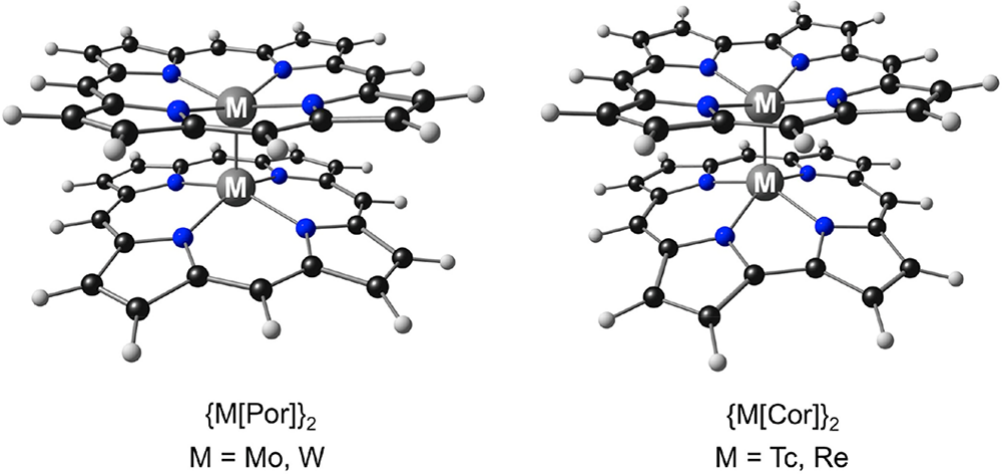
Molecules Studied in This Work

Ever since they were first postulated by Cotton over a half-century ago,^1−3^ quadruple bonds have captured the imagination of inorganic and theoretical chemists. While the classic work focused on quadruple bonds between d^4^ transition metal ions,^4−10^ exciting new avenues promise quadruple bonds involving the p-block^11−14^ and quadruple bonds involving an actinide.^15,16^ That said, key questions remain about classical quadruple-bonded systems. Many remain insufficiently characterized electrochemically, with relatively little information available on reduction potentials and HOMO–LUMO gaps.^5,6,8−10^ On the theoretical front as well, there is a general dearth of information relative to ionization potentials, electron affinities, periodic trends, and relativistic effects. The recent synthesis of rhenium corrole dimers in our laboratory allowed some of the first measurements of electrochemical HOMO–LUMO gaps for a quadruple-bonded system, which turned out to be around 1.0–1.1 eV.^17^ The question naturally arose as to how much that gap would be for ^99^Tc corrole dimers as well as for quadruple-bonded Mo and W porphyrin dimers.^18,19^ Although the rotational barriers of the latter compounds afford a certain measure of the strength of the δ-bond,^20,21^ electrochemical studies of these fascinating complexes do not appear to have been reported. Piqued by this knowledge gap, we chose to examine key properties, including S–T gaps, ionization potentials,^22−28^ and electron affinities,^29−31^ of selected quadruple-bonded metalloporphyrin and metallocorrole^32−34^ dimers by means of scalar-relativistic density functional theory (DFT) calculations. Four isoelectronic complexes were studied—unsubstituted Group 6 porphyrin dimers, {M[Por]}_2_ (M = Mo, W), and unsubstituted Group 7 corrole dimers, {M[Cor]}_2_ (M = Tc, Re; Chart 1). The results, described below, revealed a remarkably wide range of calculated properties.

## Results and Discussion

The existing theoretical literature on quadruple bonding (which includes advanced *ab initio* multiconfigurational calculations on small quadruple-bonded systems)^35^ clearly indicates generalized gradient approximation (GGA) functionals as preferable to hybrid functionals.^36,37^ We reached the same conclusions in the early stages of the present study. Thus, while B3LYP^38−41^ led to negative singlet–triplet (S–T) gaps, i.e., incorrectly predicted triplet ground states, GGA functionals such as BP86^38,42^ and OLYP^39,43^ led to generally positive adiabatic S–T gaps (Δ*E*^a^_S–T_). Dispersion-corrected, scalar-relativistic OLYP-D3^44^/ZORA^45^/STO-TZ2P calculations also yielded molecular geometries in excellent agreement with relevant crystallographic data. Thus, the optimized Mo–Mo (2.149 Å) and W–W (2.301) distances for {M[Por]}_2_ (M = Mo, W) agree well with crystallographic distances of reported for {Mo[TPP]}_2_ [2.239(1) Å]^46^ and {W[TPP]}_2_ [2.352(1) Å],^47^ respectively.^48^ Likewise, the optimized Re–Re distance (2.199 Å) is in excellent agreement with that observed [2.2364(6) Å] for Re[T*p*MePC]}_2_. For {Tc[Cor]}_2_, experimental analogues have yet to be synthesized; however, the optimized Tc–Tc distance of 2.091 Å is in similarly good agreement with Tc–Tc distances in a number of quadruple-bonded ^99^Tc complexes with nonporphyrinoid supporting ligands.^49−52^ As we calculated adiabatic ionization potentials and electron affinities of the four model compounds, however, we made a striking observation: these properties spanned a range of >1 eV across the four compounds—an astonishing finding, considering that the compounds are all charge-neutral and essentially isoelectronic. The discussion below centers largely around documenting and rationalizing this remarkable prediction.

### a Molecular Orbital Energy Level Diagrams and Qualitative Aspects of Bonding

**Figure 1:**
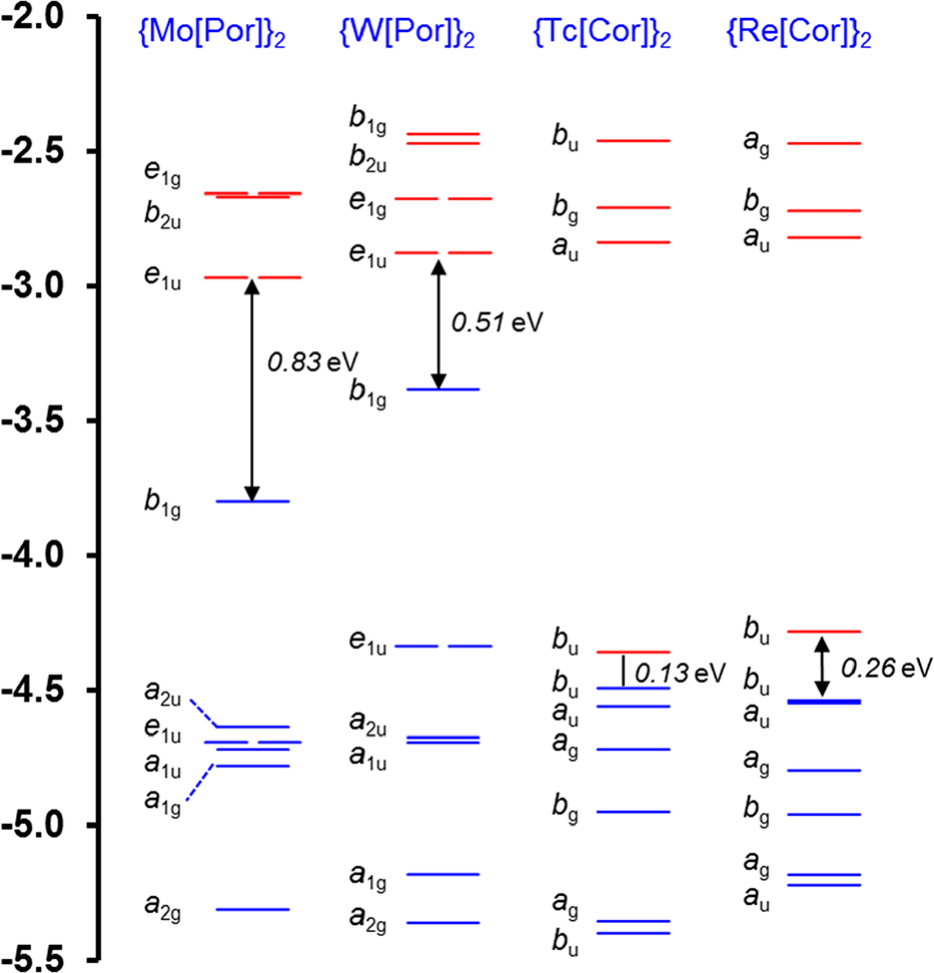
Kohn–Sham MO energy levels (eV) from OLYP-D3/ZORA/STO-TZ2P calculations. The irreps refer to the point groups *D*_4h_ and *C*_2h_ for the porphyrin and corrole derivatives, respectively.

A comparative Kohn–Sham molecular orbital (MO) energy level diagram (Figure 1) helps set the stage for a quantitative discussion of the electronic differences among the four compounds. For all the molecules studied, four of the six HOMOs may be described as in- and out-of-phase combinations of the Gouterman-type “a_1u_” and “a_2u_” HOMOs.^53−55^ Likewise, four of the six LUMOs are derived from Gouterman-type “e_g_” LUMOs. These irreducible representations refer to the *D*_4*h*_ point group of an idealized metalloporphyrin; they are also used to loosely describe the HOMOs of corrole derivatives, which are also known to follow the four-orbital model.^56^ The four molecules of interest are thus essentially isoelectronic. Yet, Figure 1 makes it abundantly clear that there are dramatic differences in the orbital energy eigenvalue spectra among the four compounds.

- Both the HOMO and LUMO energy levels are much higher for the two porphyrin dimers than for the two corrole dimers.
- For the two porphyrin dimers, the HOMOs are well-separated energetically from all other occupied MOs, but that is not the case for the corrole dimers.
- For the two corrole dimers, the very low-energy LUMOs are nondegenerate and separated by enormous energy gaps from all other unoccupied MOs; the porphyrin dimers in contrast exhibit degenerate LUMOs, with the next unoccupied MOs not very much higher in energy.

**Figure 2:**
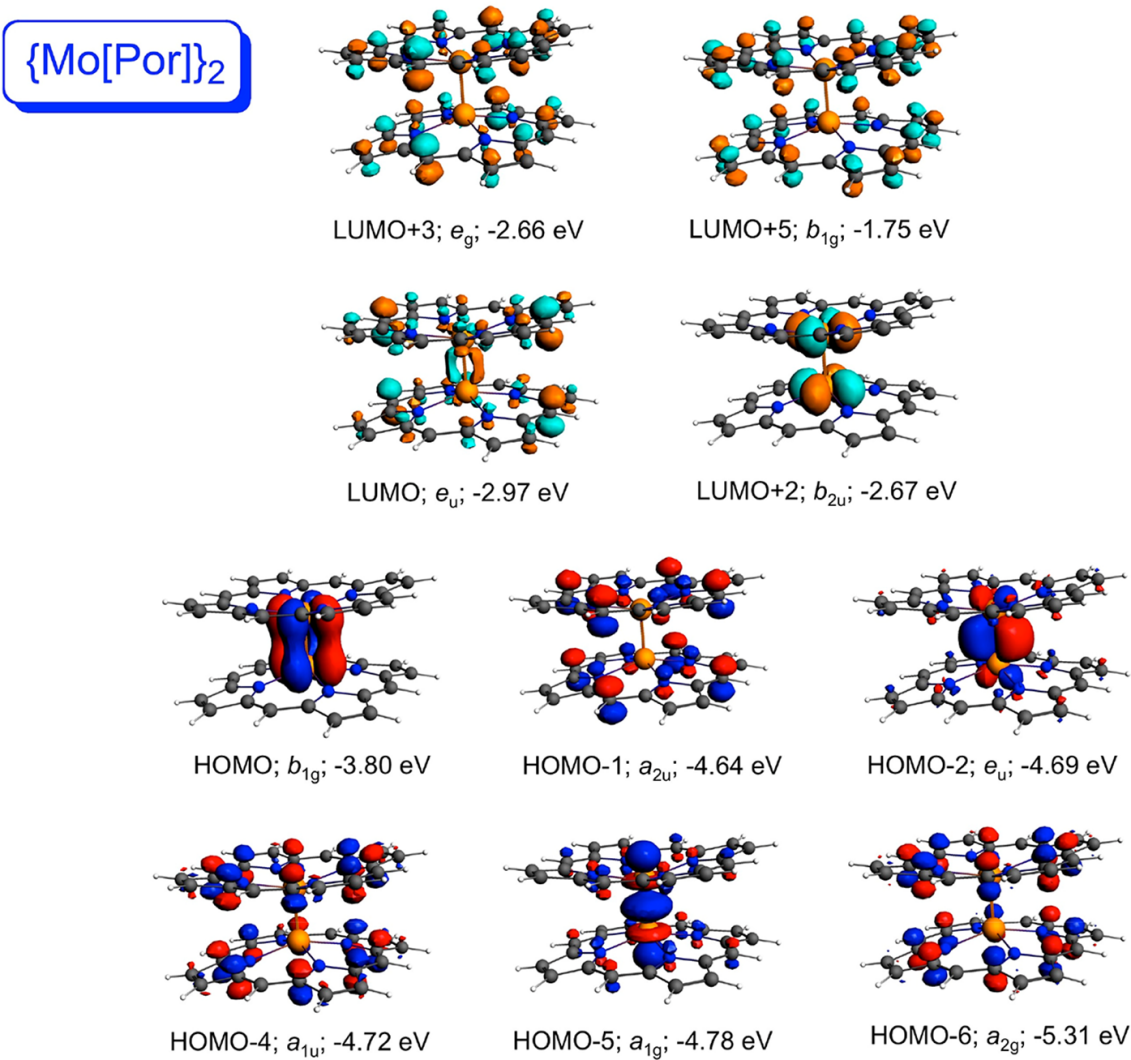
Selected frontier Kohn–Sham MOs of {Mo[Por]}_2_ (*D*_4*h*_) along with irreducible representations and orbital energies.

**Figure 3:**
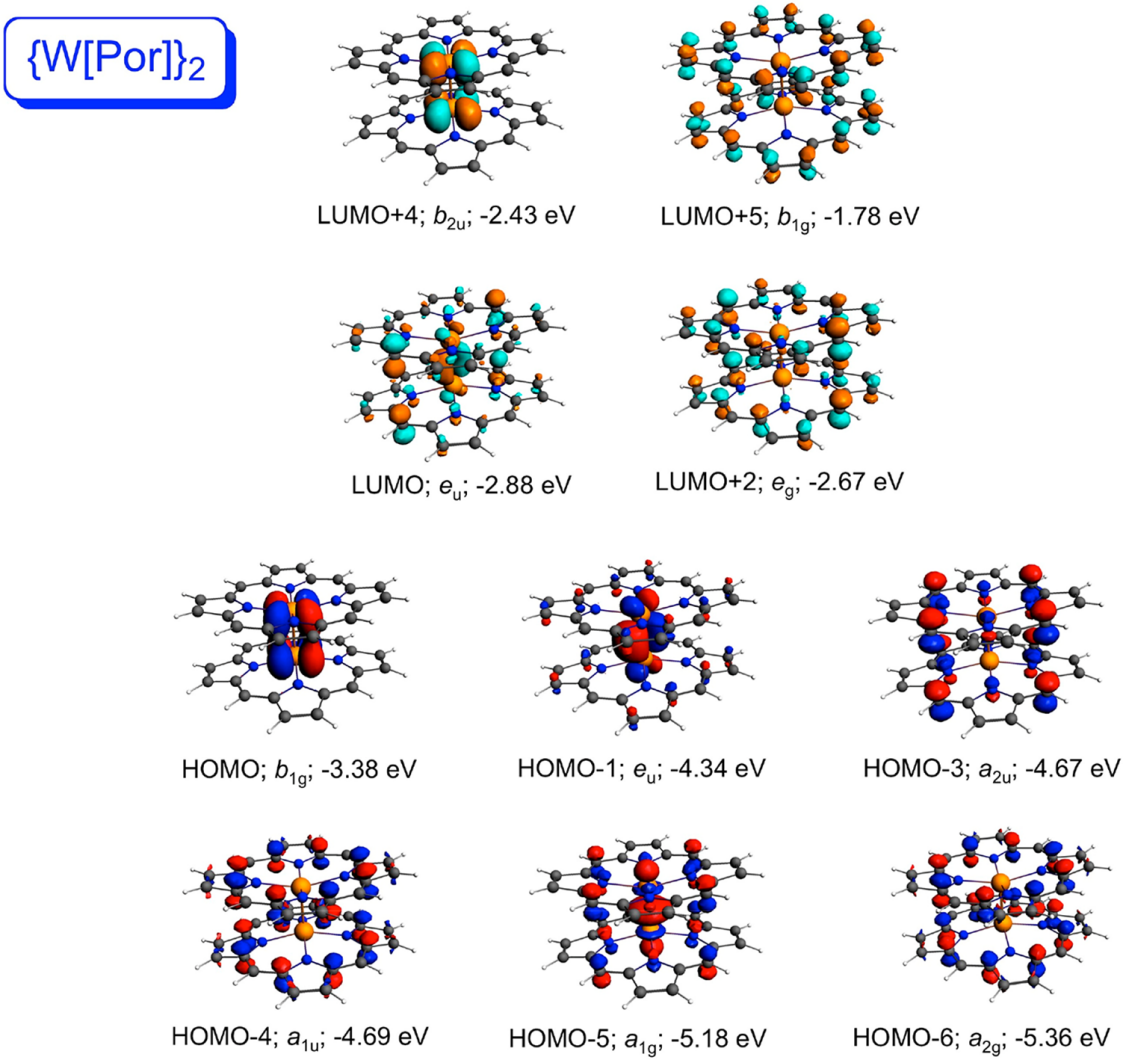
Selected frontier Kohn–Sham MOs of {W[Por]}_2_ (*D*_4*h*_) along with irreducible representations and orbital energies.

**Figure 4:**
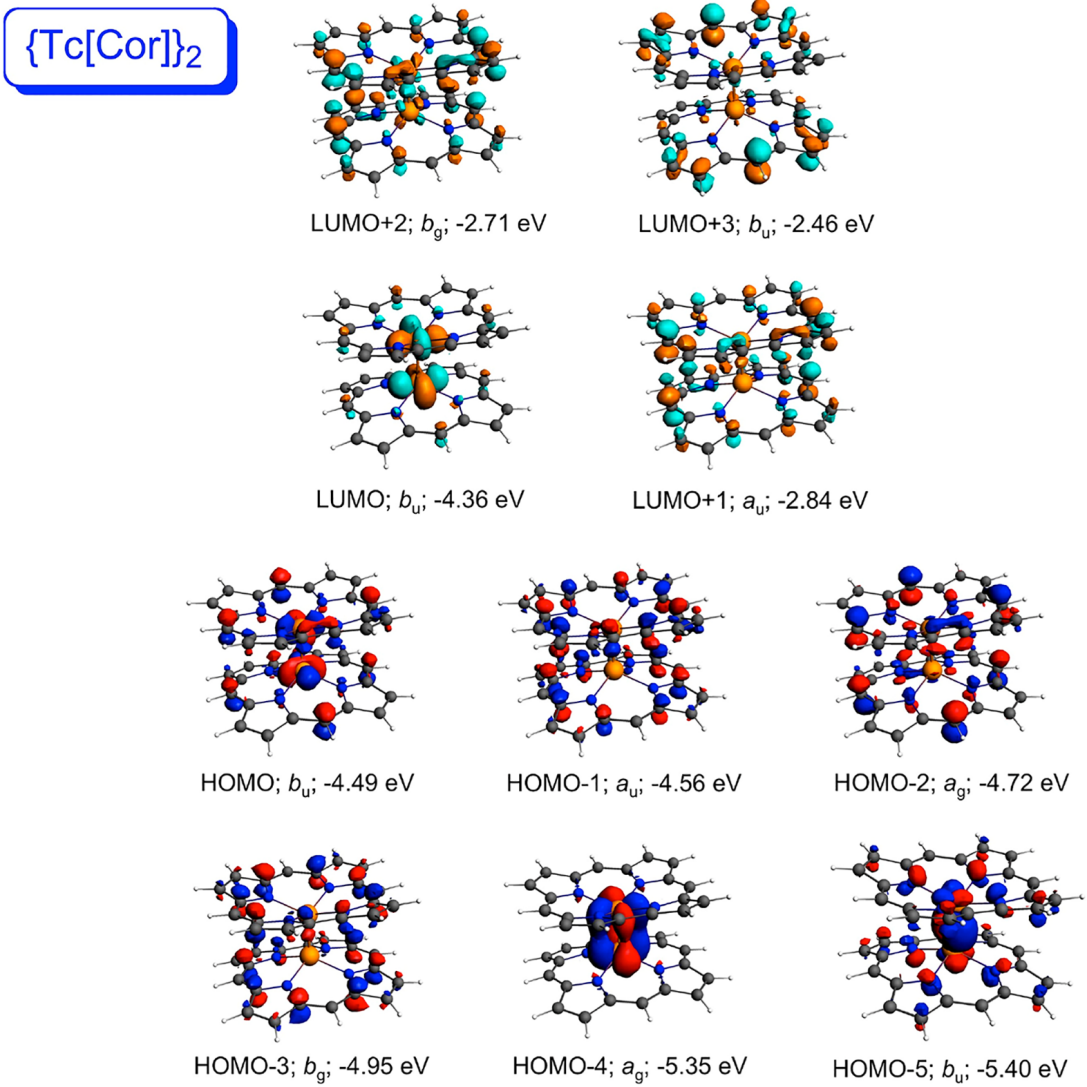
Selected frontier Kohn–Sham MOs of {Tc[Cor]}_2_ (*C*_2*h*_) along with irreducible representations and orbital energies.

**Figure 5:**
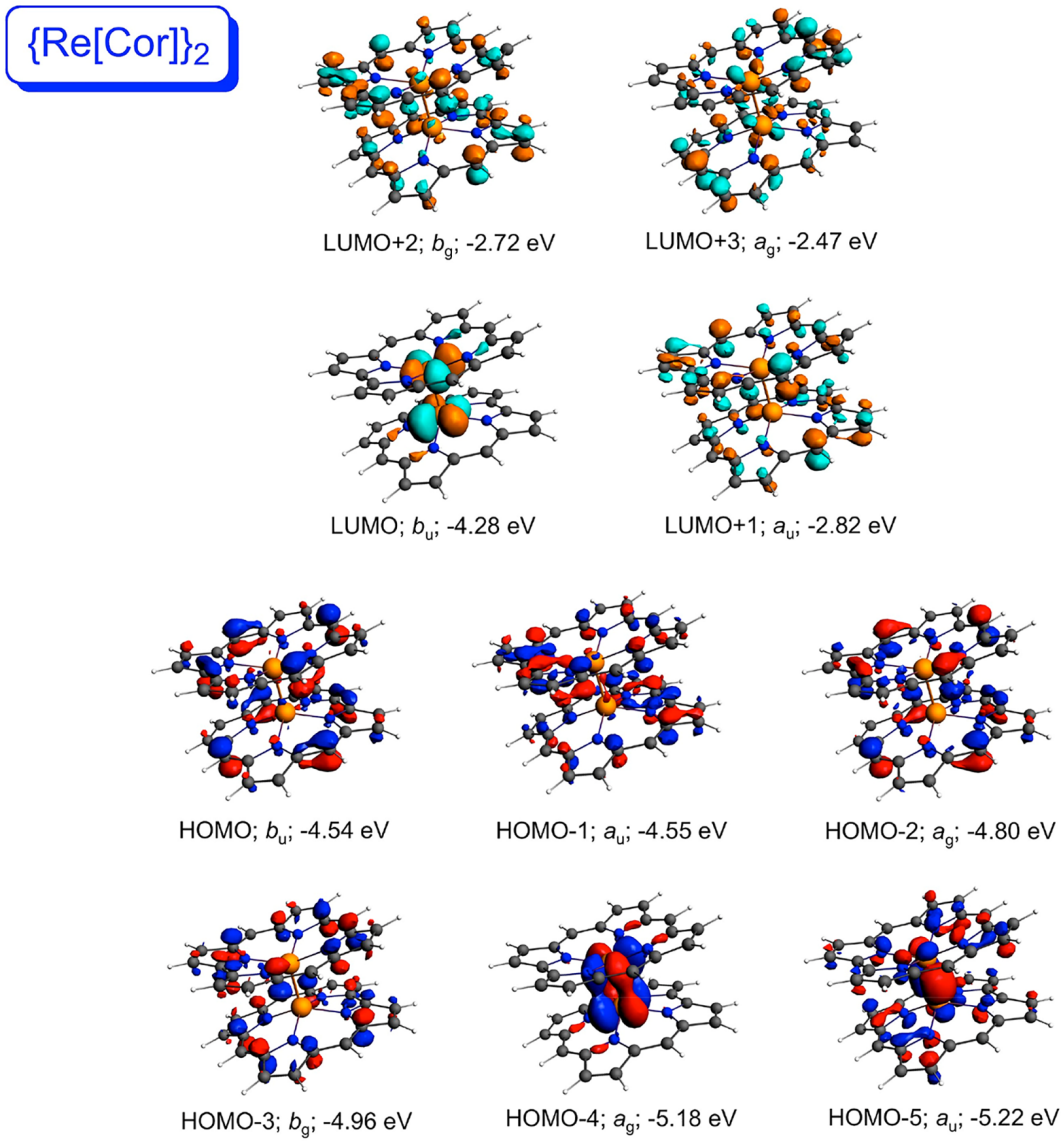
Selected frontier Kohn–Sham MOs of {Re[Cor]}_2_ (*C*_2*h*_) along with irreducible representations and orbital energies.

A visual examination of the relevant MOs goes a long way toward explaining the above differences among the compounds. For both {M[Por]}_2_ systems (Figures 2 and 3), the high-energy HOMO corresponds to the metal–metal δ-bond; the δ*-MO corresponds to the LUMO+2 in {Mo[Por]}_2_ and to the LUMO+4 in {W[Por]}_2_. By contrast, the δ- and δ*-MOs of the {M[Cor]}_2_ systems (Figures 4 and 5) are greatly depressed in energy. For both metallocorrole dimers, the δ-MO corresponds to the HOMO–4, lower in energy than all four Gouterman-type corrole-based HOMOs. Likewise, the δ*-MO is unambiguously the LUMO, with the Gouterman-type corrole-based LUMOs much higher in energy.

What accounts for the dramatic differences in δ- and δ*-orbital energies between the metalloporphyrin and metallocorrole dimers? An obvious difference centers around the oxidation state of the metal, +II for the metalloporphyrin dimers and +III for the corrole dimers. The higher metal oxidation state in the latter compounds, one might argue, results in a substantial lowering of the orbital energies of metal-based MOs, including the δ- and δ*-MOs. This argument is further examined below.

### b Ionization Potentials and Electron Affinities

As alluded to above, the most remarkable finding of this study is the sheer range of the calculated, adiabatic IPs and EAs of the four molecules studied. Both properties span a range of >1 eV (Table 1), which should also translate to a correspondingly wide range of electrochemical redox potentials.

**Table 1:** Selected Calculated Properties (in Units of eV, Where Relevant) of the Molecules Studied

compound	Δ*E*^a^_S–T_	Δ*E*^v^_S–T_	Δ*E*_HOMO–LUMO_	Δ*E*_S0–S1_	IP^a^	EA^a^	ρ^M^_Mulliken_
{Mo[Por]}_2_	0.13	0.36	0.83	0.84	5.37	1.42	1.282
{W[Por]}_2_	0.08	0.19	0.51	0.54	4.82	1.31	1.288
{Tc[Cor]}_2_	–0.01	0.00	0.13	0.23	5.57	2.81	1.392
{Re[Cor]}_2_	0.10	0.24	0.26	0.28	5.88	2.37	1.430

**Figure 6:**
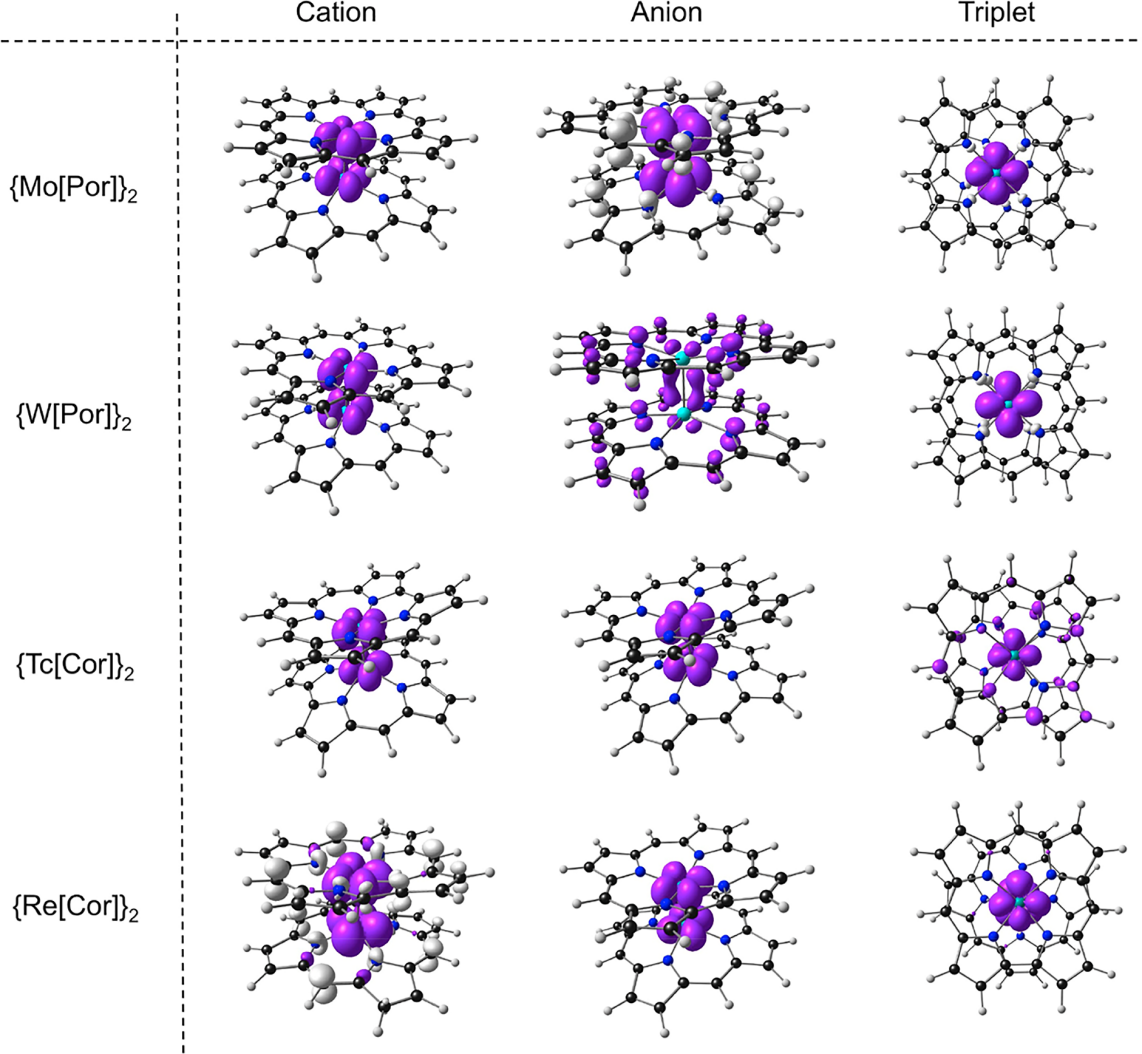
Spin density plots for the cationic, anionic, and triplet states of the molecules studied. Majority and minority spin densities are depicted in purple and ivory, respectively.

Choosing {Mo[Por]}_2_ as the reference for our discussion, the IP of 5.37 eV is considerably lower than that of free-base porphine (∼6.9 eV),^23−26^ reflecting a relatively low IP for the δ-bond. The IP of {W[Por]}_2_ (4.82 eV) is even lower, reflecting greater relativistic destabilization of the 5d orbitals in the latter complex. The IP of {Re[Cor]}_2_ (5.88 eV), in contrast, is substantially higher, by >1.0 eV relative to {W[Por]}_2_, as expected for electron removal from a corrole-based MO (Figures 2 and 6).

The EAs of the two metalloporphyrin dimers are similar, 1.36 ± 0.06 eV, and rather similar to that calculated for free-base porphine and other simple porphyrins, consistent with macrocycle-centered electron addition. In contrast, the EAs of the two metallocorrole dimers are dramatically higher, by >1.0 eV relative to {W[Por]}_2_, consistent with addition of an electron to the low-energy δ*-LUMOs.

The above differences in IPs and EAs among the four model compounds should translate to dramatic differences in electrochemical redox potentials among Group 6 (Mo and W) porphyrin dimers and Group 7 (Tc and Re) corrole dimers. Unfortunately, although Mo and W porphyrin dimers have been studied in considerable depth,^5,6,18−21^ their electrochemical characteristics apparently have not been explored. In the same vein, ^99^Tc corrole dimers have not yet been synthesized. That said, Re corrole dimers have been recently synthesized and examined via cyclic voltammetry;^17^ these measurements do indicate unusually high (in algebraic terms) reduction potentials (compared with, say, triple-bonded Ru^57−59^ and Os corrole dimers,^60^ consistent with the high calculated EAs.

The metal Mulliken charges for the four compounds suggest a plausible explanation for variations in orbital energy for metal-based δ- and δ*-MOs among the four compounds. Thus, the M(III) centers in the corrole derivatives exhibit distinctly higher Mulliken charges (by a margin of >0.1 e) than the M(II) centers in the porphyrin derivatives. In other words, the metal *oxidation state*, +III versus +II, appears to exert a *decisive* influence on δ- and δ*-orbital energies (and associated redox potentials). The same argument could also be made in terms of metal valence: the metal centers in the corrole derivatives are heptavalent, while those in the porphyrin derivatives are hexavalent (noting that homonuclear bonds do not contribute to oxidation state^61^).

As might be expected from Figure 1, the singlet–triplet gaps of the four compounds of interest span a much smaller range than either the IPs and EAs. The vertical S–T gaps range from a high of 0.36 eV for {Mo[Por]}_2_ to zero for {Tc[Cor]}_2_. The adiabatic S–T gaps are somewhat lower. In contrast, the HOMO–LUMO gaps and the TDDFT S_0_–S_1_ gaps, both evaluated at the ground-state singlet geometries, are higher than the S–T gaps. Unfortunately, as for IPs and EAs, there are few experimental measurements that are directly related to these calculated energy gaps.^4^ The key relevant quantity is the electrochemical HOMO–LUMO gap (defined as the difference between oxidation and reduction potentials) of Re corrole dimers, which, at 1.0–1.1 eV,^17^ is one of the lowest among metallocorrole derivatives,^62−64^ reflecting the unusually high reduction potentials (or EAs) of the compounds.

### c Nature of the Ionized and Triplet States

Although we have attempted to assign calculated, adiabatic IPs and EAs to processes involving individual MOs, a number of the ionized states undergo substantial geometrical relaxation.^65^ In particular, uncoupling of the δ-bond results in mutual rotation of the porphyrin and corrole ligands, i.e., to a partially or fully staggered conformation. The nature of the ionized and triplet states, in our view, is most efficiently gauged from a visual inspection of their spin density profiles (Figure 6).

As might be expected from Figure 1, the cationic states of Group 6 (Mo and W) porphyrin dimers correspond unambiguously to a δ^1^-state, with essentially *D*_4*h*_ symmetry. In the same vein, the anionic states of the Group 7 (Tc and Re) corrole dimers correspond unambiguously to a δ^*1^-state, with essentially *C*_2*h*_ symmetry, i.e., half-occupancy of the δ*-MO does not result in a symmetry lowering. In each of these four states, the electronic spin density is exclusively localized on the metal–metal axis.

Figure 1 also implies that the anionic states of the metalloporphyrin dimers and the cationic states of the metallocorrole dimers are each likely to belong to phalanx of near-degenerate states. With the present calculations, the lowest-energy state of the {Mo[Por]}_2_ anion corresponds to a triplet Mo–Mo moiety with a δ^1^δ^′1^-configuration antiferromagnetically coupled to a porphyrin radical.^66−74^ The lowest-energy state of the {W[Por]}_2_ anion, in contrast, is a purely porphyrin-based radical. Somewhat surprisingly, the lowest-energy state of the {Tc[Cor]}_2_ cation corresponds simply to a δ^1^-state, even though the δ-MO is the HOMO–4 for the corresponding neutral species. In contrast, the lowest-energy state of the {Re[Cor]}_2_ cation corresponds to a triplet Re–Re bond with a δ^1^δ^′1^ configuration antiferromagnetically coupled to a porphyrin “a_2u_”-based radical. For these states, it is quite possible that solvation, counterions, and other environmental factors may lead to the stabilization of an alternate ground state.

## Conclusions

The present study was motivated in part by the recent synthesis of Re corrole dimers.^17^ Cyclic voltammetry of these complexes revealed unexpectedly high reduction potentials and small electrochemical HOMO–LUMO gaps of 1.0–1.1 eV. To our surprise, we could not find analogous electrochemical data for the isoelectronic and otherwise well-studied Group 6 metalloporphyrin dimers. DFT calculations on {M[Por]}_2_ (M = Mo, W) were accordingly undertaken as a first step toward obtaining a unified picture of key quantitative properties of quadruple-bonded metalloporphyrin and metallocorrole dimers. To our considerable surprise, the results predicted that both the IPs and EAs of the two metallocorrole dimers should be 1 eV or more higher than the corresponding values for the metalloporphyrin dimers—a remarkable prediction considering all four species are charge-neutral. Our calculations indicate that this difference is related to the energetics of δ- and δ*-MOs of the compounds; for the two metallocorrole dimers, with +III metal centers, the orbital energies of the two highly localized MOs are dramatically lower relative to the metalloporphyrin dimers. There can be little doubt that these calculated differences among the compounds studied should also translate to dramatic differences in experimental redox potentials. The results also highlight the key role of the metal oxidation state in modulating redox potentials associated with metal–metal bonds. We hope to present an experimental follow-up to the present study in the not too distant future.

## Experimental Section

### Computational Methods

DFT calculations were carried out with the ADF 2018 program system.^75^ Relativistic effects were taken into account with the zeroth-order regular approximation (ZORA^45^) to the Dirac equation applied as a scalar correction. Specially optimized all-electron ZORA STO-TZ2P basis sets were used throughout. A variety of exchange-correlation functionals were tested, including BP86,^38,42^ B3LYP,^38−41^ TPSS, and TPSSh;^76,77^ the results quoted are those for OLYP^43,39^ (augmented with Grimme’s D3^44^ dispersion corrections), one of the better generalized gradient approximations that we have extensively tested in our studies of metalloporphyrin-type compounds.^78−82^

Throughout, molecular symmetry was used in the calculations, allowing for full analysis of molecular orbitals and electronic states in terms of their symmetry properties. Thus, the neutral metalloporphyrin and metallocorrole derivatives were found to correspond to their highest conceivable symmetries, *D*_4*h*_ and *C*_2*h*_, respectively. Ionized and triplet states were optimized with lower symmetry constraints—*D*_2_ for porphyrin derivatives and *C*_2_ for corrole derivatives (as explicitly indicated in the Supporting Information)—so as to allow for Jahn–Teller/pseudo-Jahn–Teller distortions, including rotation of the porphyrin/corrole ligands about the metal–metal axis. Care was exercised, via a combination of symmetry-unconstrained optimizations and frequency analyses, to ensure that the symmetry constraints used were not unduly restrictive.

## Supporting Information Available

Optimized Cartesian coordinates (PDF)
