# Supplementary material for: Comparing Isoelectronic, Quadruple-Bonded Metalloporphyrin and Metallocorrole Dimers: Scalar-Relativistic DFT Calculations Predict a >1 eV Range for Ionization Potential and Electron Affinity
Source: ACS Phys Chem Au. 2021 Oct 21;2(2):70–8. doi: 10.1021/acsphyschemau.1c00030 (PMC9955219; doi:10.1021/acsphyschemau.1c00030)
Supplement: Supplementary file 1 — pg1c00030_si_001.pdf [file pg1c00030_si_001.pdf]

Supporting Information

Comparing Isoelectronic, Quadruple-Bonded Metalloporphyrin and Metallocorrole Dimers: Scalar-Relativistic DFT Calculations Predict a > 1-eV Range for Ionization Potential and Electron Affinity

Jeanet Conradie,[a,b] Hugo Vazquez-Lima,[a] Abraham B. Alemayehu[a] and Abhik Ghosh*,[a]

[a] Department of Chemistry, UiT – The Arctic University of Norway, N-9037 Tromsø, Norway;
Email: abhik.ghosh@uit.no; Telephone: +47 45476145
[b]Department of Chemistry, University of the Free State, P.O. Box 339, Bloemfontein 9300, Republic of South Africa.

Optimized OLYP-D3/ZORA/STO-TZ2P Cartesian coordinates (Å)

Table of Contents 1
1. {Mo[Por]}$_2$; *S* = 0; D_{4h} 2
2. {Mo[Por]}$_2$; *S* = 1; D_2 3
3. {Mo[Por]}$_2$ cation; *S* = ½; D_2 5
4. {Mo[Por]}$_2$ anion; *S* = ½; D_2 6
5. {W[Por]}$_2$; *S* = 0; D_{4h} 8
6. {W[Por]}$_2$; *S* = 1; D_2 9
7. {W[Por]}$_2$ cation; *S* = ½; D_2 11
8. {W[Por]}$_2$ anion; *S* = ½; D_2 12
9. {Tc[Cor]}$_2$; *S* = 0; C_{2h} 14
10. {Tc[Cor]}$_2$; *S* = 1; C_2 15
11. {Tc[Cor]}$_2$ cation; *S* = ½; C_2 17
12. {Tc[Cor]}$_2$ anion; *S* = ½; C_2 18
13. {Re[Cor]}$_2$; *S* = 0; C_{2h} 20
14. {Re[Cor]}$_2$; *S* = 1; C_2 21
15. {Tc[Cor]}$_2$ cation; *S* = ½; C_2 23
16. {Re[Cor]}$_2$ anion; *S* = ½; C_2 24

Optimized OLYP/ZORA-STO-TZ2P Cartesian coordinates (Å); Spin and symmetry constraint

1. {Mo[Por]}$_2$; *S* = 0; D_{4h}

C	0.000000000	3.435318000	1.732295000
C	0.000000000	3.435318000	-1.732295000
C	0.000000000	-3.435318000	1.732295000
C	0.000000000	-3.435318000	-1.732295000
C	1.249747000	2.819086000	1.696406000
C	1.249747000	2.819086000	-1.696406000
C	1.249747000	-2.819086000	1.696406000
C	1.249747000	-2.819086000	-1.696406000
C	2.516014000	3.481563000	1.890702000
C	2.516014000	3.481563000	-1.890702000
C	2.516014000	-3.481563000	1.890702000
C	2.516014000	-3.481563000	-1.890702000
C	2.819086000	1.249747000	1.696406000
C	2.819086000	1.249747000	-1.696406000
C	2.819086000	-1.249747000	1.696406000
C	2.819086000	-1.249747000	-1.696406000
C	3.435318000	0.000000000	1.732295000
C	3.435318000	0.000000000	-1.732295000
C	3.481563000	2.516014000	1.890702000
C	3.481563000	2.516014000	-1.890702000
C	3.481563000	-2.516014000	1.890702000
C	3.481563000	-2.516014000	-1.890702000
C	-1.249747000	2.819086000	1.696406000
C	-1.249747000	2.819086000	-1.696406000
C	-1.249747000	-2.819086000	1.696406000
C	-1.249747000	-2.819086000	-1.696406000
C	-2.516014000	3.481563000	1.890702000
C	-2.516014000	3.481563000	-1.890702000
C	-2.516014000	-3.481563000	1.890702000
C	-2.516014000	-3.481563000	-1.890702000
C	-2.819086000	1.249747000	1.696406000
C	-2.819086000	1.249747000	-1.696406000
C	-2.819086000	-1.249747000	1.696406000
C	-2.819086000	-1.249747000	-1.696406000
C	-3.435318000	0.000000000	1.732295000
C	-3.435318000	0.000000000	-1.732295000
C	-3.481563000	2.516014000	1.890702000
C	-3.481563000	2.516014000	-1.890702000
C	-3.481563000	-2.516014000	1.890702000
C	-3.481563000	-2.516014000	-1.890702000
H	0.000000000	4.514390000	1.858994000
H	0.000000000	4.514390000	-1.858994000
H	0.000000000	-4.514390000	1.858994000
H	0.000000000	-4.514390000	-1.858994000

H	2.636090000	4.548641000	2.028337000
H	2.636090000	4.548641000	-2.028337000
H	2.636090000	-4.548641000	2.028337000
H	2.636090000	-4.548641000	-2.028337000
H	4.514390000	0.000000000	1.858994000
H	4.514390000	0.000000000	-1.858994000
H	4.548641000	2.636090000	2.028337000
H	4.548641000	2.636090000	-2.028337000
H	4.548641000	-2.636090000	2.028337000
H	4.548641000	-2.636090000	-2.028337000
H	-2.636090000	4.548641000	2.028337000
H	-2.636090000	4.548641000	-2.028337000
H	-2.636090000	-4.548641000	2.028337000
H	-2.636090000	-4.548641000	-2.028337000
H	-4.514390000	0.000000000	1.858994000
H	-4.514390000	0.000000000	-1.858994000
H	-4.548641000	2.636090000	2.028337000
H	-4.548641000	2.636090000	-2.028337000
H	-4.548641000	-2.636090000	2.028337000
H	-4.548641000	-2.636090000	-2.028337000
Mo	0.000000000	0.000000000	1.074262000
Mo	0.000000000	0.000000000	-1.074262000
N	1.465762000	1.465762000	1.550572000
N	1.465762000	1.465762000	-1.550572000
N	1.465762000	-1.465762000	1.550572000
N	1.465762000	-1.465762000	-1.550572000
N	-1.465762000	1.465762000	1.550572000
N	-1.465762000	1.465762000	-1.550572000
N	-1.465762000	-1.465762000	1.550572000
N	-1.465762000	-1.465762000	-1.550572000

2. {Mo[Por]}$_2$; *S* = 1; *D*$_2$

C	1.686530000	0.392436000	3.061534000
C	1.686530000	-0.392436000	-3.061534000
C	1.688594000	3.060289000	-0.391990000
C	1.688594000	-3.060289000	0.391990000
C	1.694098000	1.765411000	-2.532376000
C	1.694098000	-1.765411000	2.532376000
C	1.698299000	2.532054000	1.766121000
C	1.698299000	-2.532054000	-1.766121000
C	1.722050000	2.939845000	-1.780465000
C	1.722050000	-2.939845000	1.780465000
C	1.724514000	1.780787000	2.941021000
C	1.724514000	-1.780787000	-2.941021000
C	1.861408000	0.347186000	-4.286666000
C	1.861408000	-0.347186000	4.286666000
C	1.869254000	1.674491000	-3.960763000
C	1.869254000	-1.674491000	3.960763000
C	1.869401000	4.285123000	0.346825000

C	1.869401000	-4.285123000	-0.346825000
C	1.877807000	3.959872000	1.674289000
C	1.877807000	-3.959872000	-1.674289000
C	-1.686530000	0.392436000	-3.061534000
C	-1.686530000	-0.392436000	3.061534000
C	-1.688594000	3.060289000	0.391990000
C	-1.688594000	-3.060289000	-0.391990000
C	-1.694098000	1.765411000	2.532376000
C	-1.694098000	-1.765411000	-2.532376000
C	-1.698299000	2.532054000	-1.766121000
C	-1.698299000	-2.532054000	1.766121000
C	-1.722050000	2.939845000	1.780465000
C	-1.722050000	-2.939845000	-1.780465000
C	-1.724514000	1.780787000	-2.941021000
C	-1.724514000	-1.780787000	2.941021000
C	-1.861408000	0.347186000	4.286666000
C	-1.861408000	-0.347186000	-4.286666000
C	-1.869254000	1.674491000	3.960763000
C	-1.869254000	-1.674491000	-3.960763000
C	-1.869401000	4.285123000	-0.346825000
C	-1.869401000	-4.285123000	0.346825000
C	-1.877807000	3.959872000	-1.674289000
C	-1.877807000	-3.959872000	1.674289000
H	1.836066000	3.865030000	-2.338469000
H	1.836066000	-3.865030000	2.338469000
H	1.838947000	2.338866000	3.866100000
H	1.838947000	-2.338866000	-3.866100000
H	1.985543000	0.102780000	5.263406000
H	1.985543000	-0.102780000	-5.263406000
H	1.996814000	5.261011000	-0.104052000
H	1.996814000	-5.261011000	0.104052000
H	2.000306000	2.523903000	-4.619385000
H	2.000306000	-2.523903000	4.619385000
H	2.011631000	4.618329000	2.523614000
H	2.011631000	-4.618329000	-2.523614000
H	-1.836066000	3.865030000	2.338469000
H	-1.836066000	-3.865030000	-2.338469000
H	-1.838947000	2.338866000	-3.866100000
H	-1.838947000	-2.338866000	3.866100000
H	-1.985543000	0.102780000	-5.263406000
H	-1.985543000	-0.102780000	5.263406000
H	-1.996814000	5.261011000	0.104052000
H	-1.996814000	-5.261011000	-0.104052000
H	-2.000306000	2.523903000	4.619385000
H	-2.000306000	-2.523903000	-4.619385000
H	-2.011631000	4.618329000	-2.523614000
H	-2.011631000	-4.618329000	2.523614000
Mo	1.118121000	0.000000000	0.000000000
Mo	-1.118121000	0.000000000	0.000000000
N	1.555139000	0.496660000	-2.013591000
N	1.555139000	-0.496660000	2.013591000

N	1.556359000	2.013034000	0.497784000
N	1.556359000	-2.013034000	-0.497784000
N	-1.555139000	0.496660000	2.013591000
N	-1.555139000	-0.496660000	-2.013591000
N	-1.556359000	2.013034000	-0.497784000
N	-1.556359000	-2.013034000	0.497784000

3. {Mo[Por]}$_2$ cation; S = ½; D_2

C	1.705783000	2.930615000	0.951809000
C	1.705783000	-2.930615000	-0.951809000
C	1.708350000	1.268928000	2.806876000
C	1.708350000	-1.268928000	-2.806876000
C	1.712211000	0.951734000	-2.929705000
C	1.712211000	-0.951734000	2.929705000
C	1.714981000	2.807116000	-1.268576000
C	1.714981000	-2.807116000	1.268576000
C	1.740132000	2.560908000	2.292338000
C	1.740132000	-2.560908000	-2.292338000
C	1.750756000	2.292231000	-2.560240000
C	1.750756000	-2.292231000	2.560240000
C	1.897800000	4.264279000	0.448547000
C	1.897800000	-4.264279000	-0.448547000
C	1.899541000	0.915487000	4.187843000
C	1.899541000	-0.915487000	-4.187843000
C	1.901109000	0.447671000	-4.263418000
C	1.901109000	-0.447671000	4.263418000
C	1.904752000	4.188254000	-0.914487000
C	1.904752000	-4.188254000	0.914487000
C	-1.705783000	2.930615000	-0.951809000
C	-1.705783000	-2.930615000	0.951809000
C	-1.708350000	1.268928000	-2.806876000
C	-1.708350000	-1.268928000	2.806876000
C	-1.712211000	0.951734000	2.929705000
C	-1.712211000	-0.951734000	-2.929705000
C	-1.714981000	2.807116000	1.268576000
C	-1.714981000	-2.807116000	-1.268576000
C	-1.740132000	2.560908000	-2.292338000
C	-1.740132000	-2.560908000	2.292338000
C	-1.750756000	2.292231000	2.560240000
C	-1.750756000	-2.292231000	-2.560240000
C	-1.897800000	4.264279000	-0.448547000
C	-1.897800000	-4.264279000	0.448547000
C	-1.899541000	0.915487000	-4.187843000
C	-1.899541000	-0.915487000	4.187843000
C	-1.901109000	0.447671000	4.263418000
C	-1.901109000	-0.447671000	-4.263418000
C	-1.904752000	4.188254000	0.914487000
C	-1.904752000	-4.188254000	-0.914487000
H	1.866510000	3.364489000	3.011708000

H	1.866510000	-3.364489000	-3.011708000
H	1.881299000	3.011450000	-3.363459000
H	1.881299000	-3.011450000	3.363459000
H	2.037996000	5.138388000	1.070943000
H	2.037996000	-5.138388000	-1.070943000
H	2.041476000	1.630365000	4.987939000
H	2.041476000	-1.630365000	-4.987939000
H	2.044792000	1.069490000	-5.137364000
H	2.044792000	-1.069490000	5.137364000
H	2.051152000	4.988040000	-1.628822000
H	2.051152000	-4.988040000	1.628822000
H	-1.866510000	3.364489000	-3.011708000
H	-1.866510000	-3.364489000	3.011708000
H	-1.881299000	3.011450000	3.363459000
H	-1.881299000	-3.011450000	-3.363459000
H	-2.037996000	5.138388000	-1.070943000
H	-2.037996000	-5.138388000	1.070943000
H	-2.041476000	1.630365000	-4.987939000
H	-2.041476000	-1.630365000	4.987939000
H	-2.044792000	1.069490000	5.137364000
H	-2.044792000	-1.069490000	-5.137364000
H	-2.051152000	4.988040000	1.628822000
H	-2.051152000	-4.988040000	-1.628822000
Mo	1.098801000	0.000000000	0.000000000
Mo	-1.098801000	0.000000000	0.000000000
N	1.568758000	2.058809000	-0.113796000
N	1.568758000	-2.058809000	0.113796000
N	1.569460000	0.113467000	2.058123000
N	1.569460000	-0.113467000	-2.058123000
N	-1.568758000	2.058809000	0.113796000
N	-1.568758000	-2.058809000	-0.113796000
N	-1.569460000	0.113467000	-2.058123000
N	-1.569460000	-0.113467000	2.058123000

4. {Mo[Por]}$_2$ anion; *S* = ½; *D*$_2$

C	1.655917000	2.102229000	2.275401000
C	1.655917000	-2.102229000	-2.275401000
C	1.666178000	3.089620000	-0.028046000
C	1.666178000	-3.089620000	0.028046000
C	1.674359000	0.033417000	3.096396000
C	1.674359000	-0.033417000	-3.096396000
C	1.681907000	3.166908000	1.361119000
C	1.681907000	-3.166908000	-1.361119000
C	1.685017000	2.265318000	-2.099513000
C	1.685017000	-2.265318000	2.099513000
C	1.713671000	1.365801000	-3.157507000
C	1.713671000	-1.365801000	3.157507000
C	1.828003000	2.208443000	3.697494000
C	1.828003000	-2.208443000	-3.697494000

C	1.844159000	0.928735000	4.202404000
C	1.844159000	-0.928735000	-4.202404000
C	1.851551000	4.199903000	-0.934787000
C	1.851551000	-4.199903000	0.934787000
C	1.866928000	3.692229000	-2.203676000
C	1.866928000	-3.692229000	2.203676000
C	-1.655917000	2.102229000	-2.275401000
C	-1.655917000	-2.102229000	2.275401000
C	-1.666178000	3.089620000	0.028046000
C	-1.666178000	-3.089620000	-0.028046000
C	-1.674359000	0.033417000	-3.096396000
C	-1.674359000	-0.033417000	3.096396000
C	-1.681907000	3.166908000	-1.361119000
C	-1.681907000	-3.166908000	1.361119000
C	-1.685017000	2.265318000	2.099513000
C	-1.685017000	-2.265318000	-2.099513000
C	-1.713671000	1.365801000	3.157507000
C	-1.713671000	-1.365801000	-3.157507000
C	-1.828003000	2.208443000	-3.697494000
C	-1.828003000	-2.208443000	3.697494000
C	-1.844159000	0.928735000	-4.202404000
C	-1.844159000	-0.928735000	4.202404000
C	-1.851551000	4.199903000	0.934787000
C	-1.851551000	-4.199903000	-0.934787000
C	-1.866928000	3.692229000	2.203676000
C	-1.866928000	-3.692229000	-2.203676000
H	1.776755000	4.161798000	1.786810000
H	1.776755000	-4.161798000	-1.786810000
H	1.825167000	1.796234000	-4.149187000
H	1.825167000	-1.796234000	4.149187000
H	1.943510000	3.141561000	4.235671000
H	1.943510000	-3.141561000	-4.235671000
H	1.968075000	0.617764000	5.232886000
H	1.968075000	-0.617764000	-5.232886000
H	1.976954000	5.230316000	-0.624120000
H	1.976954000	-5.230316000	0.624120000
H	2.001961000	4.226093000	-3.136915000
H	2.001961000	-4.226093000	3.136915000
H	-1.776755000	4.161798000	-1.786810000
H	-1.776755000	-4.161798000	1.786810000
H	-1.825167000	1.796234000	4.149187000
H	-1.825167000	-1.796234000	-4.149187000
H	-1.943510000	3.141561000	-4.235671000
H	-1.943510000	-3.141561000	4.235671000
H	-1.968075000	0.617764000	-5.232886000
H	-1.968075000	-0.617764000	5.232886000
H	-1.976954000	5.230316000	0.624120000
H	-1.976954000	-5.230316000	-0.624120000
H	-2.001961000	4.226093000	3.136915000
H	-2.001961000	-4.226093000	-3.136915000
Mo	1.111265000	0.000000000	0.000000000

Mo	-1.111265000	0.000000000	0.000000000
N	1.533234000	1.931009000	-0.765184000
N	1.533234000	-1.931009000	0.765184000
N	1.536402000	0.772137000	1.935968000
N	1.536402000	-0.772137000	-1.935968000
N	-1.533234000	1.931009000	0.765184000
N	-1.533234000	-1.931009000	-0.765184000
N	-1.536402000	0.772137000	-1.935968000
N	-1.536402000	-0.772137000	1.935968000

5. {W[Por]}$_2$; S = 0; D_{4h}

C	0.000000000	3.437301000	1.699965000
C	0.000000000	3.437301000	-1.699965000
C	0.000000000	-3.437301000	1.699965000
C	0.000000000	-3.437301000	-1.699965000
C	1.245090000	2.819117000	1.669494000
C	1.245090000	2.819117000	-1.669494000
C	1.245090000	-2.819117000	1.669494000
C	1.245090000	-2.819117000	-1.669494000
C	2.513772000	3.479877000	1.814743000
C	2.513772000	3.479877000	-1.814743000
C	2.513772000	-3.479877000	1.814743000
C	2.513772000	-3.479877000	-1.814743000
C	2.819117000	1.245090000	1.669494000
C	2.819117000	1.245090000	-1.669494000
C	2.819117000	-1.245090000	1.669494000
C	2.819117000	-1.245090000	-1.669494000
C	3.437301000	0.000000000	1.699965000
C	3.437301000	0.000000000	-1.699965000
C	3.479877000	2.513772000	1.814743000
C	3.479877000	2.513772000	-1.814743000
C	3.479877000	-2.513772000	1.814743000
C	3.479877000	-2.513772000	-1.814743000
C	-1.245090000	2.819117000	1.669494000
C	-1.245090000	2.819117000	-1.669494000
C	-1.245090000	-2.819117000	1.669494000
C	-1.245090000	-2.819117000	-1.669494000
C	-2.513772000	3.479877000	1.814743000
C	-2.513772000	3.479877000	-1.814743000
C	-2.513772000	-3.479877000	1.814743000
C	-2.513772000	-3.479877000	-1.814743000
C	-2.819117000	1.245090000	1.669494000
C	-2.819117000	1.245090000	-1.669494000
C	-2.819117000	-1.245090000	1.669494000
C	-2.819117000	-1.245090000	-1.669494000
C	-3.437301000	0.000000000	1.699965000
C	-3.437301000	0.000000000	-1.699965000
C	-3.479877000	2.513772000	1.814743000

C	-3.479877000	2.513772000	-1.814743000
C	-3.479877000	-2.513772000	1.814743000
C	-3.479877000	-2.513772000	-1.814743000
H	0.000000000	4.519373000	1.796525000
H	0.000000000	4.519373000	-1.796525000
H	0.000000000	-4.519373000	1.796525000
H	0.000000000	-4.519373000	-1.796525000
H	2.638331000	4.550289000	1.916813000
H	2.638331000	4.550289000	-1.916813000
H	2.638331000	-4.550289000	1.916813000
H	2.638331000	-4.550289000	-1.916813000
H	4.519373000	0.000000000	1.796525000
H	4.519373000	0.000000000	-1.796525000
H	4.550289000	2.638331000	1.916813000
H	4.550289000	2.638331000	-1.916813000
H	4.550289000	-2.638331000	1.916813000
H	4.550289000	-2.638331000	-1.916813000
H	-2.638331000	4.550289000	1.916813000
H	-2.638331000	4.550289000	-1.916813000
H	-2.638331000	-4.550289000	1.916813000
H	-2.638331000	-4.550289000	-1.916813000
H	-4.519373000	0.000000000	1.796525000
H	-4.519373000	0.000000000	-1.796525000
H	-4.550289000	2.638331000	1.916813000
H	-4.550289000	2.638331000	-1.916813000
H	-4.550289000	-2.638331000	1.916813000
H	-4.550289000	-2.638331000	-1.916813000
N	1.453024000	1.453024000	1.554540000
N	1.453024000	1.453024000	-1.554540000
N	1.453024000	-1.453024000	1.554540000
N	1.453024000	-1.453024000	-1.554540000
N	-1.453024000	1.453024000	1.554540000
N	-1.453024000	1.453024000	-1.554540000
N	-1.453024000	-1.453024000	1.554540000
N	-1.453024000	-1.453024000	-1.554540000
W	0.000000000	0.000000000	1.150650000
W	0.000000000	0.000000000	-1.150650000

6. {W[Por]}$_2$; *S* = 1; *D*$_2$

C	1.689465000	0.933749000	2.937360000
C	1.689465000	-0.933749000	-2.937360000
C	1.693076000	1.290245000	-2.800192000
C	1.693076000	-1.290245000	2.800192000
C	1.696220000	2.936615000	-0.934159000
C	1.696220000	-2.936615000	0.934159000
C	1.700392000	2.798982000	1.289881000
C	1.700392000	-2.798982000	-1.289881000
C	1.722495000	2.577586000	-2.276480000
C	1.722495000	-2.577586000	2.276480000

C	1.722976000	2.275700000	2.577594000
C	1.722976000	-2.275700000	-2.577594000
C	1.825335000	0.420101000	4.272108000
C	1.825335000	-0.420101000	-4.272108000
C	1.829531000	0.943855000	-4.187641000
C	1.829531000	-0.943855000	4.187641000
C	1.843134000	4.270169000	-0.420401000
C	1.843134000	-4.270169000	0.420401000
C	1.846884000	4.185501000	0.943617000
C	1.846884000	-4.185501000	-0.943617000
C	-1.689465000	0.933749000	-2.937360000
C	-1.689465000	-0.933749000	2.937360000
C	-1.693076000	1.290245000	2.800192000
C	-1.693076000	-1.290245000	-2.800192000
C	-1.696220000	2.936615000	0.934159000
C	-1.696220000	-2.936615000	-0.934159000
C	-1.700392000	2.798982000	-1.289881000
C	-1.700392000	-2.798982000	1.289881000
C	-1.722495000	2.577586000	2.276480000
C	-1.722495000	-2.577586000	-2.276480000
C	-1.722976000	2.275700000	-2.577594000
C	-1.722976000	-2.275700000	2.577594000
C	-1.825335000	0.420101000	-4.272108000
C	-1.825335000	-0.420101000	4.272108000
C	-1.829531000	0.943855000	4.187641000
C	-1.829531000	-0.943855000	-4.187641000
C	-1.843134000	4.270169000	0.420401000
C	-1.843134000	-4.270169000	-0.420401000
C	-1.846884000	4.185501000	-0.943617000
C	-1.846884000	-4.185501000	0.943617000
H	1.816766000	3.389307000	-2.992183000
H	1.816766000	-3.389307000	2.992183000
H	1.817494000	2.991464000	3.389221000
H	1.817494000	-2.991464000	-3.389221000
H	1.920026000	1.035272000	5.157676000
H	1.920026000	-1.035272000	-5.157676000
H	1.927412000	1.662915000	-4.990934000
H	1.927412000	-1.662915000	4.990934000
H	1.943712000	5.155178000	-1.035499000
H	1.943712000	-5.155178000	1.035499000
H	1.951432000	4.987956000	1.662861000
H	1.951432000	-4.987956000	-1.662861000
H	-1.816766000	3.389307000	2.992183000
H	-1.816766000	-3.389307000	-2.992183000
H	-1.817494000	2.991464000	-3.389221000
H	-1.817494000	-2.991464000	3.389221000
H	-1.920026000	1.035272000	-5.157676000
H	-1.920026000	-1.035272000	5.157676000
H	-1.927412000	1.662915000	4.990934000
H	-1.927412000	-1.662915000	-4.990934000
H	-1.943712000	5.155178000	1.035499000

H	-1.943712000	-5.155178000	-1.035499000
H	-1.951432000	4.987956000	-1.662861000
H	-1.951432000	-4.987956000	1.662861000
N	1.583445000	0.128900000	-2.048162000
N	1.583445000	-0.128900000	2.048162000
N	1.586164000	2.047712000	0.128398000
N	1.586164000	-2.047712000	-0.128398000
N	-1.583445000	0.128900000	2.048162000
N	-1.583445000	-0.128900000	-2.048162000
N	-1.586164000	2.047712000	-0.128398000
N	-1.586164000	-2.047712000	0.128398000
W	1.199091000	0.000000000	0.000000000
W	-1.199091000	0.000000000	0.000000000

7. {W[Por]}$_2$ cation; *S* = ½; *D*$_2$

C	1.687738000	1.108847000	2.873478000
C	1.687738000	-1.108847000	-2.873478000
C	1.688503000	2.868837000	1.120405000
C	1.688503000	-2.868837000	-1.120405000
C	1.702857000	1.119100000	-2.867777000
C	1.702857000	-1.119100000	2.867777000
C	1.703406000	2.872429000	-1.107612000
C	1.703406000	-2.872429000	1.107612000
C	1.716150000	2.426325000	2.436143000
C	1.716150000	-2.426325000	-2.436143000
C	1.740321000	2.434539000	-2.424809000
C	1.740321000	-2.434539000	2.424809000
C	1.848814000	0.675123000	4.232570000
C	1.848814000	-0.675123000	-4.232570000
C	1.853989000	4.229248000	0.692153000
C	1.853989000	-4.229248000	-0.692153000
C	1.859778000	0.689713000	-4.228897000
C	1.859778000	-0.689713000	4.228897000
C	1.863147000	4.231469000	-0.672678000
C	1.863147000	-4.231469000	0.672678000
C	-1.687738000	1.108847000	-2.873478000
C	-1.687738000	-1.108847000	2.873478000
C	-1.688503000	2.868837000	-1.120405000
C	-1.688503000	-2.868837000	1.120405000
C	-1.702857000	1.119100000	2.867777000
C	-1.702857000	-1.119100000	-2.867777000
C	-1.703406000	2.872429000	1.107612000
C	-1.703406000	-2.872429000	-1.107612000
C	-1.716150000	2.426325000	-2.436143000
C	-1.716150000	-2.426325000	2.436143000
C	-1.740321000	2.434539000	2.424809000
C	-1.740321000	-2.434539000	-2.424809000
C	-1.848814000	0.675123000	-4.232570000
C	-1.848814000	-0.675123000	4.232570000

C	-1.853989000	4.229248000	-0.692153000
C	-1.853989000	-4.229248000	0.692153000
C	-1.859778000	0.689713000	4.228897000
C	-1.859778000	-0.689713000	-4.228897000
C	-1.863147000	4.231469000	0.672678000
C	-1.863147000	-4.231469000	-0.672678000
H	1.823217000	3.188879000	3.201680000
H	1.823217000	-3.188879000	-3.201680000
H	1.857337000	3.199269000	-3.186725000
H	1.857337000	-3.199269000	3.186725000
H	1.964301000	1.342776000	5.076261000
H	1.964301000	-1.342776000	-5.076261000
H	1.972184000	5.069946000	1.363080000
H	1.972184000	-5.069946000	-1.363080000
H	1.986138000	1.359876000	-5.069092000
H	1.986138000	-1.359876000	5.069092000
H	1.990770000	5.074325000	-1.339190000
H	1.990770000	-5.074325000	1.339190000
H	-1.823217000	3.188879000	-3.201680000
H	-1.823217000	-3.188879000	3.201680000
H	-1.857337000	3.199269000	3.186725000
H	-1.857337000	-3.199269000	-3.186725000
H	-1.964301000	1.342776000	-5.076261000
H	-1.964301000	-1.342776000	5.076261000
H	-1.972184000	5.069946000	-1.363080000
H	-1.972184000	-5.069946000	1.363080000
H	-1.986138000	1.359876000	5.069092000
H	-1.986138000	-1.359876000	-5.069092000
H	-1.990770000	5.074325000	1.339190000
H	-1.990770000	-5.074325000	-1.339190000
N	1.574380000	0.003703000	-2.049372000
N	1.574380000	-0.003703000	2.049372000
N	1.574831000	2.049315000	0.004413000
N	1.574831000	-2.049315000	-0.004413000
N	-1.574380000	0.003703000	2.049372000
N	-1.574380000	-0.003703000	-2.049372000
N	-1.574831000	2.049315000	-0.004413000
N	-1.574831000	-2.049315000	0.004413000
W	1.155951000	0.000000000	0.000000000
W	-1.155951000	0.000000000	0.000000000

8. {W[Por]}$_2$ anion; *S* = ½; *D*$_2$

C	1.645201000	1.111464000	2.883577000
C	1.645201000	-1.111464000	-2.883577000
C	1.651411000	1.119729000	-2.879146000
C	1.651411000	-1.119729000	2.879146000
C	1.663727000	2.878761000	1.120339000
C	1.663727000	-2.878761000	-1.120339000
C	1.670471000	2.881628000	-1.109571000

C	1.670471000	-2.881628000	1.109571000
C	1.671974000	2.435530000	2.436137000
C	1.671974000	-2.435530000	-2.436137000
C	1.682281000	2.442238000	-2.426979000
C	1.682281000	-2.442238000	2.426979000
C	1.774016000	0.681397000	4.241688000
C	1.774016000	-0.681397000	-4.241688000
C	1.778969000	0.693973000	-4.238946000
C	1.778969000	-0.693973000	4.238946000
C	1.808991000	4.245107000	0.691234000
C	1.808991000	-4.245107000	-0.691234000
C	1.813881000	4.246902000	-0.675714000
C	1.813881000	-4.246902000	0.675714000
C	-1.645201000	1.111464000	-2.883577000
C	-1.645201000	-1.111464000	2.883577000
C	-1.651411000	1.119729000	2.879146000
C	-1.651411000	-1.119729000	-2.879146000
C	-1.663727000	2.878761000	-1.120339000
C	-1.663727000	-2.878761000	1.120339000
C	-1.670471000	2.881628000	1.109571000
C	-1.670471000	-2.881628000	-1.109571000
C	-1.671974000	2.435530000	-2.436137000
C	-1.671974000	-2.435530000	2.436137000
C	-1.682281000	2.442238000	2.426979000
C	-1.682281000	-2.442238000	-2.426979000
C	-1.774016000	0.681397000	-4.241688000
C	-1.774016000	-0.681397000	4.241688000
C	-1.778969000	0.693973000	4.238946000
C	-1.778969000	-0.693973000	-4.238946000
C	-1.808991000	4.245107000	-0.691234000
C	-1.808991000	-4.245107000	0.691234000
C	-1.813881000	4.246902000	0.675714000
C	-1.813881000	-4.246902000	-0.675714000
H	1.748000000	3.201171000	3.203618000
H	1.748000000	-3.201171000	-3.203618000
H	1.762794000	3.210017000	-3.191875000
H	1.762794000	-3.210017000	3.191875000
H	1.853534000	1.348630000	5.090897000
H	1.853534000	-1.348630000	-5.090897000
H	1.863402000	1.364068000	-5.085554000
H	1.863402000	-1.364068000	5.085554000
H	1.896980000	5.090954000	1.362250000
H	1.896980000	-5.090954000	-1.362250000
H	1.906166000	5.094575000	-1.343741000
H	1.906166000	-5.094575000	1.343741000
H	-1.748000000	3.201171000	-3.203618000
H	-1.748000000	-3.201171000	3.203618000
H	-1.762794000	3.210017000	3.191875000
H	-1.762794000	-3.210017000	-3.191875000
H	-1.853534000	1.348630000	-5.090897000
H	-1.853534000	-1.348630000	5.090897000

H	-1.863402000	1.364068000	5.085554000
H	-1.863402000	-1.364068000	-5.085554000
H	-1.896980000	5.090954000	-1.362250000
H	-1.896980000	-5.090954000	1.362250000
H	-1.906166000	5.094575000	1.343741000
H	-1.906166000	-5.094575000	-1.343741000
N	1.544748000	0.002714000	-2.060366000
N	1.544748000	-0.002714000	2.060366000
N	1.562655000	2.066432000	0.003981000
N	1.562655000	-2.066432000	-0.003981000
N	-1.544748000	0.002714000	2.060366000
N	-1.544748000	-0.002714000	-2.060366000
N	-1.562655000	2.066432000	-0.003981000
N	-1.562655000	-2.066432000	0.003981000
W	1.140103000	0.000000000	0.000000000
W	-1.140103000	0.000000000	0.000000000

9. {Tc[Cor]}$_2$; *S* = 0; *C*$_{2h}$

C	0.128332000	1.984922000	2.815612000
C	0.128332000	1.984922000	-2.815612000
C	0.760805000	-2.880405000	3.498897000
C	0.760805000	-2.880405000	-3.498897000
C	1.179689000	-2.996309000	1.264571000
C	1.179689000	-2.996309000	-1.264571000
C	1.241885000	1.298014000	3.345415000
C	1.241885000	1.298014000	-3.345415000
C	1.551357000	-3.500597000	2.556752000
C	1.551357000	-3.500597000	-2.556752000
C	1.657735000	-3.385904000	0.000000000
C	2.254199000	0.654277000	2.610536000
C	2.254199000	0.654277000	-2.610536000
C	3.375377000	0.111658000	0.717813000
C	3.375377000	0.111658000	-0.717813000
C	3.572433000	0.224217000	2.976218000
C	3.572433000	0.224217000	-2.976218000
C	4.262897000	-0.094053000	1.809757000
C	4.262897000	-0.094053000	-1.809757000
C	-0.128332000	-1.984922000	2.815612000
C	-0.128332000	-1.984922000	-2.815612000
C	-0.760805000	2.880405000	3.498897000
C	-0.760805000	2.880405000	-3.498897000
C	-1.179689000	2.996309000	1.264571000
C	-1.179689000	2.996309000	-1.264571000
C	-1.241885000	-1.298014000	3.345415000
C	-1.241885000	-1.298014000	-3.345415000
C	-1.551357000	3.500597000	2.556752000
C	-1.551357000	3.500597000	-2.556752000
C	-1.657735000	3.385904000	0.000000000
C	-2.254199000	-0.654277000	2.610536000

C	-2.254199000	-0.654277000	-2.610536000
C	-3.375377000	-0.111658000	0.717813000
C	-3.375377000	-0.111658000	-0.717813000
C	-3.572433000	-0.224217000	2.976218000
C	-3.572433000	-0.224217000	-2.976218000
C	-4.262897000	0.094053000	1.809757000
C	-4.262897000	0.094053000	-1.809757000
H	0.757010000	-3.055125000	4.567719000
H	0.757010000	-3.055125000	-4.567719000
H	1.392436000	1.390178000	4.417636000
H	1.392436000	1.390178000	-4.417636000
H	2.299399000	-4.264477000	2.727842000
H	2.299399000	-4.264477000	-2.727842000
H	2.457899000	-4.120944000	0.000000000
H	3.961696000	0.191517000	3.986167000
H	3.961696000	0.191517000	-3.986167000
H	5.293257000	-0.418345000	1.737612000
H	5.293257000	-0.418345000	-1.737612000
H	-0.757010000	3.055125000	4.567719000
H	-0.757010000	3.055125000	-4.567719000
H	-1.392436000	-1.390178000	4.417636000
H	-1.392436000	-1.390178000	-4.417636000
H	-2.299399000	4.264477000	2.727842000
H	-2.299399000	4.264477000	-2.727842000
H	-2.457899000	4.120944000	0.000000000
H	-3.961696000	-0.191517000	3.986167000
H	-3.961696000	-0.191517000	-3.986167000
H	-5.293257000	0.418345000	1.737612000
H	-5.293257000	0.418345000	-1.737612000
N	0.186798000	-2.050251000	1.466752000
N	0.186798000	-2.050251000	-1.466752000
N	2.167083000	0.518609000	1.245061000
N	2.167083000	0.518609000	-1.245061000
N	-0.186798000	2.050251000	1.466752000
N	-0.186798000	2.050251000	-1.466752000
N	-2.167083000	-0.518609000	1.245061000
N	-2.167083000	-0.518609000	-1.245061000
Tc	0.589347000	0.863343000	0.000000000
Tc	-0.589347000	-0.863343000	0.000000000

10. {Tc[Cor]}$_2$; *S* = 1; *C*$_2$

C	0.240127000	-2.016172000	-3.267634000
C	0.586427000	1.739722000	3.339183000
C	0.657659000	-2.053449000	2.767728000
C	1.052897000	1.608850000	-2.916199000
C	1.313523000	-2.276568000	-2.378996000
C	1.753847000	1.375731000	2.633293000
C	1.814070000	-2.730806000	3.308443000
C	2.081511000	-2.501228000	1.066836000

C	2.217084000	1.546868000	-3.759865000
C	2.264889000	-2.564741000	-0.346924000
C	2.551691000	-2.977280000	-2.559537000
C	2.676438000	-3.013393000	2.262241000
C	2.840113000	1.150383000	-1.609995000
C	3.086740000	1.219886000	3.159340000
C	3.128507000	-3.161676000	-1.304836000
C	3.157265000	1.037629000	0.891518000
C	3.306017000	1.271689000	-2.966115000
C	3.604295000	0.970720000	-0.432982000
C	3.939164000	1.013363000	2.102935000
C	-0.240127000	2.016172000	-3.267634000
C	-0.586427000	-1.739722000	3.339183000
C	-0.657659000	2.053449000	2.767728000
C	-1.052897000	-1.608850000	-2.916199000
C	-1.313523000	2.276568000	-2.378996000
C	-1.753847000	-1.375731000	2.633293000
C	-1.814070000	2.730806000	3.308443000
C	-2.081511000	2.501228000	1.066836000
C	-2.217084000	-1.546868000	-3.759865000
C	-2.264889000	2.564741000	-0.346924000
C	-2.551691000	2.977280000	-2.559537000
C	-2.676438000	3.013393000	2.262241000
C	-2.840113000	-1.150383000	-1.609995000
C	-3.086740000	-1.219886000	3.159340000
C	-3.128507000	3.161676000	-1.304836000
C	-3.157265000	-1.037629000	0.891518000
C	-3.306017000	-1.271689000	-2.966115000
C	-3.604295000	-0.970720000	-0.432982000
C	-3.939164000	-1.013363000	2.102935000
H	0.401712000	-2.248212000	-4.316922000
H	0.694084000	1.876465000	4.411291000
H	1.946276000	-3.003764000	4.348147000
H	2.212706000	1.731514000	-4.827016000
H	2.935403000	-3.337397000	-3.505773000
H	3.346139000	1.291092000	4.208308000
H	3.613497000	-3.551780000	2.322961000
H	4.045916000	-3.694085000	-1.088172000
H	4.339837000	1.182861000	-3.275386000
H	4.672229000	0.829315000	-0.573603000
H	5.013401000	0.881989000	2.140657000
H	-0.401712000	2.248212000	-4.316922000
H	-0.694084000	-1.876465000	4.411291000
H	-1.946276000	3.003764000	4.348147000
H	-2.212706000	-1.731514000	-4.827016000
H	-2.935403000	3.337397000	-3.505773000
H	-3.346139000	-1.291092000	4.208308000
H	-3.613497000	3.551780000	2.322961000
H	-4.045916000	3.694085000	-1.088172000
H	-4.339837000	-1.182861000	-3.275386000
H	-4.672229000	-0.829315000	-0.573603000

H	-5.013401000	-0.881989000	2.140657000
N	0.897376000	-1.887589000	1.430073000
N	1.205466000	-2.002366000	-1.045194000
N	1.475728000	1.318906000	-1.620115000
N	1.834833000	1.226963000	1.261222000
N	-0.897376000	1.887589000	1.430073000
N	-1.205466000	2.002366000	-1.045194000
N	-1.475728000	-1.318906000	-1.620115000
N	-1.834833000	-1.226963000	1.261222000
Tc	0.247718000	1.019984000	-0.010611000
Tc	-0.247718000	-1.019984000	-0.010611000

11. {Tc[Cor]}$_2$ cation; *S* = ½; *C*$_2$

C	0.174329000	-2.150762000	-3.272671000
C	0.519694000	1.646483000	3.325351000
C	0.713900000	-1.961790000	2.737652000
C	1.100228000	1.720245000	-2.920148000
C	1.271181000	-2.384945000	-2.384077000
C	1.701492000	1.304903000	2.626733000
C	1.899304000	-2.610053000	3.284330000
C	2.120094000	-2.448743000	1.032264000
C	2.281587000	-2.567489000	-0.370794000
C	2.287756000	1.698602000	-3.743066000
C	2.492558000	-3.122280000	-2.559478000
C	2.743781000	-2.915377000	2.242002000
C	2.857990000	1.211857000	-1.596125000
C	3.028541000	1.127254000	3.176457000
C	3.106946000	-3.236419000	-1.318231000
C	3.138191000	1.020779000	0.903882000
C	3.356059000	1.397271000	-2.939724000
C	3.608138000	0.991866000	-0.407664000
C	3.898013000	0.953548000	2.134827000
C	-0.174329000	2.150762000	-3.272671000
C	-0.519694000	-1.646483000	3.325351000
C	-0.713900000	1.961790000	2.737652000
C	-1.100228000	-1.720245000	-2.920148000
C	-1.271181000	2.384945000	-2.384077000
C	-1.701492000	-1.304903000	2.626733000
C	-1.899304000	2.610053000	3.284330000
C	-2.120094000	2.448743000	1.032264000
C	-2.281587000	2.567489000	-0.370794000
C	-2.287756000	-1.698602000	-3.743066000
C	-2.492558000	3.122280000	-2.559478000
C	-2.743781000	2.915377000	2.242002000
C	-2.857990000	-1.211857000	-1.596125000
C	-3.028541000	-1.127254000	3.176457000
C	-3.106946000	3.236419000	-1.318231000
C	-3.138191000	-1.020779000	0.903882000
C	-3.356059000	-1.397271000	-2.939724000

C	-3.608138000	-0.991866000	-0.407664000
C	-3.898013000	-0.953548000	2.134827000
H	0.316477000	-2.437556000	-4.311184000
H	0.607064000	1.755101000	4.401797000
H	2.051104000	-2.843099000	4.330757000
H	2.306690000	1.939113000	-4.798525000
H	2.839288000	-3.547091000	-3.492770000
H	3.266912000	1.168977000	4.231568000
H	3.687715000	-3.440753000	2.302847000
H	4.020241000	-3.773651000	-1.098035000
H	4.397976000	1.335032000	-3.226336000
H	4.678780000	0.862734000	-0.536235000
H	4.970977000	0.820029000	2.188520000
H	-0.316477000	2.437556000	-4.311184000
H	-0.607064000	-1.755101000	4.401797000
H	-2.051104000	2.843099000	4.330757000
H	-2.306690000	-1.939113000	-4.798525000
H	-2.839288000	3.547091000	-3.492770000
H	-3.266912000	-1.168977000	4.231568000
H	-3.687715000	3.440753000	2.302847000
H	-4.020241000	3.773651000	-1.098035000
H	-4.397976000	-1.335032000	-3.226336000
H	-4.678780000	-0.862734000	-0.536235000
H	-4.970977000	-0.820029000	2.188520000
N	0.932321000	-1.829108000	1.397715000
N	1.203525000	-2.021594000	-1.078183000
N	1.500005000	1.366983000	-1.622646000
N	1.806559000	1.205269000	1.254778000
N	-0.932321000	1.829108000	1.397715000
N	-1.203525000	2.021594000	-1.078183000
N	-1.500005000	-1.366983000	-1.622646000
N	-1.806559000	-1.205269000	1.254778000
Tc	0.243231000	1.021771000	-0.047289000
Tc	-0.243231000	-1.021771000	-0.047289000

12. {Tc[Cor]}$_2$ anion; *S* = ½; *C*$_2$

C	0.100766000	1.906510000	-3.332250000
C	0.225943000	1.898462000	3.336228000
C	0.956926000	-2.183180000	2.624744000
C	1.055612000	-2.186533000	-2.577039000
C	1.350227000	1.524187000	-2.818998000
C	1.454505000	1.514323000	2.776473000
C	2.143863000	-2.891701000	3.004852000
C	2.185623000	-2.549909000	0.761518000
C	2.212410000	-2.550575000	-0.667458000
C	2.266234000	-2.877152000	-2.913019000
C	2.596678000	1.428915000	-3.528951000
C	2.726267000	1.412288000	3.439404000
C	2.892752000	-3.124834000	1.852641000

C	2.968653000	-3.112257000	-1.732039000
C	2.985580000	1.109759000	-1.311423000
C	3.031706000	1.102456000	1.207687000
C	3.592411000	1.176545000	-2.613370000
C	3.619202000	0.983291000	-0.063279000
C	3.686320000	1.159650000	2.486858000
C	-0.100766000	-1.906510000	-3.332250000
C	-0.225943000	-1.898462000	3.336228000
C	-0.956926000	2.183180000	2.624744000
C	-1.055612000	2.186533000	-2.577039000
C	-1.350227000	-1.524187000	-2.818998000
C	-1.454505000	-1.514323000	2.776473000
C	-2.143863000	2.891701000	3.004852000
C	-2.185623000	2.549909000	0.761518000
C	-2.212410000	2.550575000	-0.667458000
C	-2.266234000	2.877152000	-2.913019000
C	-2.596678000	-1.428915000	-3.528951000
C	-2.726267000	-1.412288000	3.439404000
C	-2.892752000	3.124834000	1.852641000
C	-2.968653000	3.112257000	-1.732039000
C	-2.985580000	-1.109759000	-1.311423000
C	-3.031706000	-1.102456000	1.207687000
C	-3.592411000	-1.176545000	-2.613370000
C	-3.619202000	-0.983291000	-0.063279000
C	-3.686320000	-1.159650000	2.486858000
H	0.055020000	2.092519000	-4.402255000
H	0.221032000	2.086450000	4.406875000
H	2.380437000	-3.222731000	4.009252000
H	2.548982000	-3.196740000	-3.909028000
H	2.706724000	1.568962000	-4.598033000
H	2.875951000	1.545484000	4.504565000
H	3.826730000	-3.669730000	1.787686000
H	3.904326000	-3.648939000	-1.632014000
H	4.653197000	1.068253000	-2.806196000
H	4.696698000	0.843259000	-0.083226000
H	4.752761000	1.044716000	2.640076000
H	-0.055020000	-2.092519000	-4.402255000
H	-0.221032000	-2.086450000	4.406875000
H	-2.380437000	3.222731000	4.009252000
H	-2.548982000	3.196740000	-3.909028000
H	-2.706724000	-1.568962000	-4.598033000
H	-2.875951000	-1.545484000	4.504565000
H	-3.826730000	3.669730000	1.787686000
H	-3.904326000	3.648939000	-1.632014000
H	-4.653197000	-1.068253000	-2.806196000
H	-4.696698000	-0.843259000	-0.083226000
H	-4.752761000	-1.044716000	2.640076000
N	1.052570000	-1.951655000	1.277126000
N	1.094840000	-1.961611000	-1.225433000
N	1.626058000	1.285913000	-1.478914000
N	1.679482000	1.281601000	1.426030000

N	-1.052570000	1.951655000	1.277126000
N	-1.094840000	1.961611000	-1.225433000
N	-1.626058000	-1.285913000	-1.478914000
N	-1.679482000	-1.281601000	1.426030000
Tc	0.235955000	1.024266000	-0.000789000
Tc	-0.235955000	-1.024266000	-0.000789000

13. {Re[Cor]}$_2$; *S* = 0; C_{2h}

C	0.154028000	2.038259000	2.819067000
C	0.154028000	2.038259000	-2.819067000
C	0.762458000	-2.908675000	3.492654000
C	0.762458000	-2.908675000	-3.492654000
C	1.199152000	-2.999179000	1.260095000
C	1.199152000	-2.999179000	-1.260095000
C	1.262711000	1.362343000	3.346543000
C	1.262711000	1.362343000	-3.346543000
C	1.581296000	-3.490269000	2.550995000
C	1.581296000	-3.490269000	-2.550995000
C	1.685140000	-3.377085000	0.000000000
C	2.253206000	0.699184000	2.602999000
C	2.253206000	0.699184000	-2.602999000
C	3.344930000	0.077801000	0.711884000
C	3.344930000	0.077801000	-0.711884000
C	3.550087000	0.215549000	2.966923000
C	3.550087000	0.215549000	-2.966923000
C	4.221306000	-0.152388000	1.803630000
C	4.221306000	-0.152388000	-1.803630000
C	-0.154028000	-2.038259000	2.819067000
C	-0.154028000	-2.038259000	-2.819067000
C	-0.762458000	2.908675000	3.492654000
C	-0.762458000	2.908675000	-3.492654000
C	-1.199152000	2.999179000	1.260095000
C	-1.199152000	2.999179000	-1.260095000
C	-1.262711000	-1.362343000	3.346543000
C	-1.262711000	-1.362343000	-3.346543000
C	-1.581296000	3.490269000	2.550995000
C	-1.581296000	3.490269000	-2.550995000
C	-1.685140000	3.377085000	0.000000000
C	-2.253206000	-0.699184000	2.602999000
C	-2.253206000	-0.699184000	-2.602999000
C	-3.344930000	-0.077801000	0.711884000
C	-3.344930000	-0.077801000	-0.711884000
C	-3.550087000	-0.215549000	2.966923000
C	-3.550087000	-0.215549000	-2.966923000
C	-4.221306000	0.152388000	1.803630000
C	-4.221306000	0.152388000	-1.803630000
H	0.761301000	-3.089816000	4.560296000
H	0.761301000	-3.089816000	-4.560296000
H	1.415609000	1.443863000	4.418854000

H	1.415609000	1.443863000	-4.418854000
H	2.364583000	-4.218086000	2.720775000
H	2.364583000	-4.218086000	-2.720775000
H	2.496785000	-4.098852000	0.000000000
H	3.940944000	0.186784000	3.976036000
H	3.940944000	0.186784000	-3.976036000
H	5.236873000	-0.520179000	1.732769000
H	5.236873000	-0.520179000	-1.732769000
H	-0.761301000	3.089816000	4.560296000
H	-0.761301000	3.089816000	-4.560296000
H	-1.415609000	-1.443863000	4.418854000
H	-1.415609000	-1.443863000	-4.418854000
H	-2.364583000	4.218086000	2.720775000
H	-2.364583000	4.218086000	-2.720775000
H	-2.496785000	4.098852000	0.000000000
H	-3.940944000	-0.186784000	3.976036000
H	-3.940944000	-0.186784000	-3.976036000
H	-5.236873000	0.520179000	1.732769000
H	-5.236873000	0.520179000	-1.732769000
N	0.165249000	-2.085437000	1.462155000
N	0.165249000	-2.085437000	-1.462155000
N	2.154012000	0.553479000	1.235284000
N	2.154012000	0.553479000	-1.235284000
N	-0.165249000	2.085437000	1.462155000
N	-0.165249000	2.085437000	-1.462155000
N	-2.154012000	-0.553479000	1.235284000
N	-2.154012000	-0.553479000	-1.235284000
Re	0.590273000	0.927732000	0.000000000
Re	-0.590273000	-0.927732000	0.000000000

14. {Re[Cor]}$_2$; *S* = 1; *C*$_2$

C	0.153073000	1.824906000	2.982548000
C	0.300636000	1.832102000	-2.983901000
C	0.830148000	-2.392807000	3.854383000
C	1.039792000	-2.243082000	-2.954300000
C	1.205449000	1.820514000	-4.095035000
C	1.497457000	1.469322000	3.188159000
C	1.669163000	-2.389007000	1.745885000
C	1.822986000	-2.422787000	-1.800335000
C	1.939551000	-2.748427000	3.091676000
C	2.208992000	-2.552402000	0.438363000
C	2.375918000	1.289697000	-2.216063000
C	2.434048000	1.177084000	2.186586000
C	2.460072000	1.493424000	-3.631797000
C	3.079145000	-3.075461000	-1.591500000
C	3.307304000	-3.161245000	-0.219773000
C	3.330419000	0.990342000	0.098132000
C	3.409772000	1.033822000	-1.302136000
C	3.847252000	0.984696000	2.315346000

C	4.389907000	0.873573000	1.054242000
C	-0.153073000	-1.824906000	2.982548000
C	-0.300636000	-1.832102000	-2.983901000
C	-0.830148000	2.392807000	3.854383000
C	-1.039792000	2.243082000	-2.954300000
C	-1.205449000	-1.820514000	-4.095035000
C	-1.497457000	-1.469322000	3.188159000
C	-1.669163000	2.389007000	1.745885000
C	-1.822986000	2.422787000	-1.800335000
C	-1.939551000	2.748427000	3.091676000
C	-2.208992000	2.552402000	0.438363000
C	-2.375918000	-1.289697000	-2.216063000
C	-2.434048000	-1.177084000	2.186586000
C	-2.460072000	-1.493424000	-3.631797000
C	-3.079145000	3.075461000	-1.591500000
C	-3.307304000	3.161245000	-0.219773000
C	-3.330419000	-0.990342000	0.098132000
C	-3.409772000	-1.033822000	-1.302136000
C	-3.847252000	-0.984696000	2.315346000
C	-4.389907000	-0.873573000	1.054242000
H	0.697450000	-2.556215000	4.916448000
H	0.928332000	2.070038000	-5.111705000
H	1.479045000	-2.531313000	-3.904810000
H	1.872446000	1.500655000	4.206890000
H	2.836018000	-3.239909000	3.446926000
H	3.375003000	1.425143000	-4.206938000
H	3.710597000	-3.471902000	-2.376699000
H	4.150379000	-3.636380000	0.264995000
H	4.377803000	0.967673000	3.259167000
H	4.402003000	0.911723000	-1.726693000
H	5.434132000	0.745519000	0.797998000
H	-0.697450000	2.556215000	4.916448000
H	-0.928332000	-2.070038000	-5.111705000
H	-1.479045000	2.531313000	-3.904810000
H	-1.872446000	-1.500655000	4.206890000
H	-2.836018000	3.239909000	3.446926000
H	-3.375003000	-1.425143000	-4.206938000
H	-3.710597000	3.471902000	-2.376699000
H	-4.150379000	3.636380000	0.264995000
H	-4.377803000	-0.967673000	3.259167000
H	-4.402003000	-0.911723000	-1.726693000
H	-5.434132000	-0.745519000	0.797998000
N	0.413626000	-1.798254000	1.725495000
N	1.040103000	1.467151000	-1.857679000
N	1.351525000	-2.081729000	-0.548862000
N	2.145129000	1.137595000	0.819662000
N	-0.413626000	1.798254000	1.725495000
N	-1.040103000	-1.467151000	-1.857679000
N	-1.351525000	2.081729000	-0.548862000
N	-2.145129000	-1.137595000	0.819662000
Re	0.318090000	1.076279000	-0.016374000

Re	-0.318090000	-1.076279000	-0.016374000

15. {Tc[Cor]}$_2$ cation; *S* = ½; *C*$_2$

C	0.067058000	1.883586000	2.975748000
C	0.379821000	1.763498000	-2.986328000
C	0.937983000	-2.479995000	3.808961000
C	0.967064000	-2.183502000	-2.997859000
C	1.305341000	1.719212000	-4.075289000
C	1.401444000	1.538714000	3.221854000
C	1.713449000	-2.427386000	1.679784000
C	1.779250000	-2.387215000	-1.874056000
C	2.021310000	-2.824959000	3.007871000
C	2.221207000	-2.567016000	0.345291000
C	2.373942000	1.225164000	2.246133000
C	2.425587000	1.224984000	-2.157716000
C	2.548555000	1.391411000	-3.572828000
C	3.036516000	-3.054292000	-1.713292000
C	3.298331000	-3.172832000	-0.350682000
C	3.324251000	0.986242000	0.194580000
C	3.435290000	0.989607000	-1.206406000
C	3.781687000	1.050775000	2.422966000
C	4.359835000	0.906623000	1.177736000
C	-0.067058000	-1.883586000	2.975748000
C	-0.379821000	-1.763498000	-2.986328000
C	-0.937983000	2.479995000	3.808961000
C	-0.967064000	2.183502000	-2.997859000
C	-1.305341000	-1.719212000	-4.075289000
C	-1.401444000	-1.538714000	3.221854000
C	-1.713449000	2.427386000	1.679784000
C	-1.779250000	2.387215000	-1.874056000
C	-2.021310000	2.824959000	3.007871000
C	-2.221207000	2.567016000	0.345291000
C	-2.373942000	-1.225164000	2.246133000
C	-2.425587000	-1.224984000	-2.157716000
C	-2.548555000	-1.391411000	-3.572828000
C	-3.036516000	3.054292000	-1.713292000
C	-3.298331000	3.172832000	-0.350682000
C	-3.324251000	-0.986242000	0.194580000
C	-3.435290000	-0.989607000	-1.206406000
C	-3.781687000	-1.050775000	2.422966000
C	-4.359835000	-0.906623000	1.177736000
H	0.831611000	-2.672957000	4.868878000
H	1.057105000	1.951419000	-5.103001000
H	1.376789000	-2.459412000	-3.965143000
H	1.754327000	1.606035000	4.246912000
H	2.921521000	-3.334793000	3.324621000
H	3.476858000	1.309489000	-4.123587000
H	3.640959000	-3.442978000	-2.522692000

H	4.146522000	-3.669153000	0.102208000
H	4.288263000	1.071729000	3.379388000
H	4.438675000	0.860838000	-1.602294000
H	5.412349000	0.786904000	0.954803000
H	-0.831611000	2.672957000	4.868878000
H	-1.057105000	-1.951419000	-5.103001000
H	-1.376789000	2.459412000	-3.965143000
H	-1.754327000	-1.606035000	4.246912000
H	-2.921521000	3.334793000	3.324621000
H	-3.476858000	-1.309489000	-4.123587000
H	-3.640959000	3.442978000	-2.522692000
H	-4.146522000	3.669153000	0.102208000
H	-4.288263000	-1.071729000	3.379388000
H	-4.438675000	-0.860838000	-1.602294000
H	-5.412349000	-0.786904000	0.954803000
N	0.467919000	-1.829477000	1.702424000
N	1.083638000	1.423321000	-1.834987000
N	1.342165000	-2.069843000	-0.600550000
N	2.118989000	1.149448000	0.879581000
N	-0.467919000	1.829477000	1.702424000
N	-1.083638000	-1.423321000	-1.834987000
N	-1.342165000	2.069843000	-0.600550000
N	-2.118989000	-1.149448000	0.879581000
Re	0.313491000	1.075686000	-0.004727000
Re	-0.313491000	-1.075686000	-0.004727000

16. {Re[Cor]}$_2$ anion; *S* = ½; *C*$_2$

C	0.150483000	1.926117000	-3.333669000
C	0.194194000	1.898644000	3.328984000
C	0.980596000	-2.188206000	2.608097000
C	1.015556000	-2.207908000	-2.595793000
C	1.389796000	1.527666000	-2.808469000
C	1.426468000	1.503814000	2.784882000
C	2.181323000	-2.875264000	2.980173000
C	2.190662000	-2.566727000	0.729756000
C	2.200775000	-2.570874000	-0.698804000
C	2.219417000	-2.901411000	-2.946130000
C	2.641035000	1.408017000	-3.503767000
C	2.686436000	1.375584000	3.462853000
C	2.916091000	-3.117134000	1.819729000
C	2.939356000	-3.132671000	-1.774185000
C	3.004229000	1.072392000	-1.284033000
C	3.020405000	1.059927000	1.235426000
C	3.619187000	0.931064000	-0.028798000
C	3.622442000	1.128089000	-2.580026000
C	3.655345000	1.103182000	2.523776000
C	-0.150483000	-1.926117000	-3.333669000
C	-0.194194000	-1.898644000	3.328984000
C	-0.980596000	2.188206000	2.608097000

C	-1.015556000	2.207908000	-2.595793000
C	-1.389796000	-1.527666000	-2.808469000
C	-1.426468000	-1.503814000	2.784882000
C	-2.181323000	2.875264000	2.980173000
C	-2.190662000	2.566727000	0.729756000
C	-2.200775000	2.570874000	-0.698804000
C	-2.219417000	2.901411000	-2.946130000
C	-2.641035000	-1.408017000	-3.503767000
C	-2.686436000	-1.375584000	3.462853000
C	-2.916091000	3.117134000	1.819729000
C	-2.939356000	3.132671000	-1.774185000
C	-3.004229000	-1.072392000	-1.284033000
C	-3.020405000	-1.059927000	1.235426000
C	-3.619187000	-0.931064000	-0.028798000
C	-3.622442000	-1.128089000	-2.580026000
C	-3.655345000	-1.103182000	2.523776000
H	0.121080000	2.119621000	-4.403131000
H	0.177690000	2.081192000	4.400629000
H	2.438896000	-3.186762000	3.985497000
H	2.488208000	-3.224183000	-3.944852000
H	2.765048000	1.545767000	-4.571602000
H	2.824451000	1.502378000	4.530342000
H	3.856520000	-3.650756000	1.751075000
H	3.877103000	-3.668497000	-1.688088000
H	4.681318000	0.992492000	-2.765352000
H	4.694327000	0.771582000	-0.036384000
H	4.716341000	0.964414000	2.694041000
H	-0.121080000	-2.119621000	-4.403131000
H	-0.177690000	-2.081192000	4.400629000
H	-2.438896000	3.186762000	3.985497000
H	-2.488208000	3.224183000	-3.944852000
H	-2.765048000	-1.545767000	-4.571602000
H	-2.824451000	-1.502378000	4.530342000
H	-3.856520000	3.650756000	1.751075000
H	-3.877103000	3.668497000	-1.688088000
H	-4.681318000	-0.992492000	-2.765352000
H	-4.694327000	-0.771582000	-0.036384000
H	-4.716341000	-0.964414000	2.694041000
N	1.054284000	-1.972880000	1.252446000
N	1.072442000	-1.979291000	-1.241609000
N	1.646732000	1.278841000	-1.462558000
N	1.665688000	1.267053000	1.433497000
N	-1.054284000	1.972880000	1.252446000
N	-1.072442000	1.979291000	-1.241609000
N	-1.646732000	-1.278841000	-1.462558000
N	-1.665688000	-1.267053000	1.433497000
Re	0.250376000	1.078600000	-0.005652000
Re	-0.250376000	-1.078600000	-0.005652000
